# Assessment of Antimicrobial Consumption in Multi-Field Hospitals with Pediatric Inpatients: Conventional vs. Novel Pediatric-Adjusted Methodologies

**DOI:** 10.3390/antibiotics12071162

**Published:** 2023-07-07

**Authors:** Svetlana Rachina, Yuliya Belkova, Roman Kozlov, Vladimir Mladov, Vladimir Mishchenko, Alla Andreeva, Olga Domanskaya, Ulyana Portnjagina, Anastasiia Dushina, Khadizhat Zainalabidova

**Affiliations:** 1 Hospital Therapy Department No. 2, I.M. Sechenov First Moscow State Medical University, 119435 Moscow, Russia; rachina_s_a@staff.sechenov.ru (S.R.); xad2001@yandex.ru (K.Z.); 2 Department of Clinical Pharmacology, Smolensk State Medical University, 214019 Smolensk, Russia; yuliya.belkova@antibiotic.ru; 3 Institute of Antimicrobial Chemotherapy, Smolensk State Medical University, 214019 Smolensk, Russia; 4 Faculty of Applied Mathematics and Control Processes, St. Petersburg State University, 199034 St. Petersburg, Russia; vmladov@outlook.com; 5 Federal Centre of Traumatology, Orthopedics and Endoprosthesis Replacement, 214019 Smolensk, Russia; vladimir.mishchenko@orthosmolensk.ru; 6 Smolensk Regional Clinical Hospital, 214018 Smolensk, Russia; alla.andreeva@antibiotic.ru; 7 Kuzbas Children’s Clinical Hospital n.a. Professor Y.E. Malachovskiy, 654063 Novokuznetsk, Russia; olga-domanskaya@mail.ru; 8 Department of Internal Medicine and General Medical Practice (Family Medicine), North-Eastern Federal University, 677007 Yakutsk, Russia; ulyana-nsk@mail.ru; 9 Institute of Engineering Physics for Biomedicine, National Research Nuclear University MEPhI (Moscow Engineering Physics Institute), 115409 Moscow, Russia; dushina02@gmail.com

**Keywords:** antimicrobial agent, consumption, pediatric, hospital, ATC/DDD methodology, defined daily dose, DDD

## Abstract

Background: the objective of this study was to propose a methodology for the assessment of antimicrobial consumption (AMC) in pediatric inpatients and to estimate variances in consumption levels in multi-field hospitals with pediatric inpatients, calculated by means of the pediatric-adjusted methodology vs. the conventional methodology. Methods: the pediatric-adjusted methodology based on the conventional ATC/DDD method and children’s DDDs (cDDD) for antimicrobials were proposed and validated in a series of probabilistic sensitivity analyses of real clinical data extracted from the receipt notes of three multi-field hospitals. Differences in AMC in multi-field hospitals with pediatric inpatients, calculated by means of the proposed methodology vs. the conventional methodology, were assessed for a virtual cohort of inpatients, with the pediatric share increasing by 1%. Results: in children ≤12 years old, assessment by the standard methodology resulted in a 59% underestimation of AMC from the levels based on prescribed doses, vs. a 25% underestimation for the proposed methodology. In a mixed-age virtual population of inpatients, the underestimation of consumption levels rose to 321% for the ATC/DDD methodology compared to the proposed one. Conclusions: the proposed methodology demonstrated a higher accuracy of AMC estimates compared to the conventional one and can be considered for the quantification of antimicrobial utilization in pediatric institutions and multi-field hospitals with a substantial share of pediatric inpatients.

## 1. Introduction

Antimicrobials (AMs) are an essential class of drugs, but rapidly increasing global antimicrobial resistance compromises their effectiveness [1,2,3]. The main driver of resistance is the misuse and overuse of AMs; responsible prescribing of these medications should be ensured in every healthcare institution [4,5]. Antimicrobial consumption (AMC) is an essential metric for designing tailored antibiotic stewardship programs and for estimating their impact on drug utilization and resistance selection. It is also of value for target setting by policy makers [6].

A World Health Organization (WHO)-recommended ATC/DDD system has been developed as a universal and easily applicable method for drug consumption assessment and benchmarking. The methodology uses the Anatomical Therapeutic Chemical Classification System (ATC), as well as the defined daily dose (DDD), with the latter being the assumed average maintenance dose per day for a drug used for its main indication in adults with a body weight of 70 kg [7]. DDD is a technical unit of consumption measurement and may differ from the recommended and prescribed daily doses (PDD) based on a patient’s individual characteristics, such as age, weight, and the type and severity of disease, and pharmacokinetic considerations.

The ATC/DDD system represents a stable drug utilization metric that enables comparisons between regions and health care institutions and allows us to examine trends in drug use over time. On the other hand, this methodology has a number of inherent limitations, one of which is the incorrect assessment of drug consumption in patients with special dosing approaches, such as critically ill patients, patients with renal or hepatic impairment, and children; inaccuracies occur in the latter group due to diversity in body weight and age-dependent dose variations. The miscalculation of AMC in such populations can severely impact our assessment of resistance selection risks and unfavorably influence drug utilization practices.

Over the last twenty years, attempts have been made to optimize consumption assessments in the pediatric population, such as by modifying the conventional ATC/DDD methodology and using different dose measurement units (PDD; days of treatment, DOT, etc.) [8,9,10,11,12,13,14], but the issue is still far from being resolved. The problem is aggravated by the uncertainty of the extent of consumption estimation errors due to pediatric inpatients’ presence in healthcare institutions.

The objective of this study was to propose a methodology for the assessment of antimicrobial consumption in pediatric inpatients and to estimate variances in consumption levels in multi-field hospitals with pediatric inpatients, calculated by means of the pediatric-adjusted methodology vs. the conventional methodology.

## 2. Results

### 2.1. Validation of the Proposed Pediatric-Adjusted Methodology

AMC assessment by means of the proposed methodology vs. the conventional ATC/DDD methodology was performed on the real clinical data from three multi-field hospitals (a total of 503,252 bed days; share of patients aged 1 month to 12 years—18.2%). The input parameter values for the proposed methodology (calculated DDD of AMs for the children of different age groups, children’s DDDs (cDDD), mean body weight, and body surface area (BSA) values) are presented in the Supplementary Tables S1, S2, and S3, respectively.

The total consumption levels were 51.38 DID for the proposed methodology vs. 48.23 DID for the conventional ATC/DDD methodology. Detailed data related to different AM consumption levels are presented in Supplementary Table S4.

**Figure 1:**
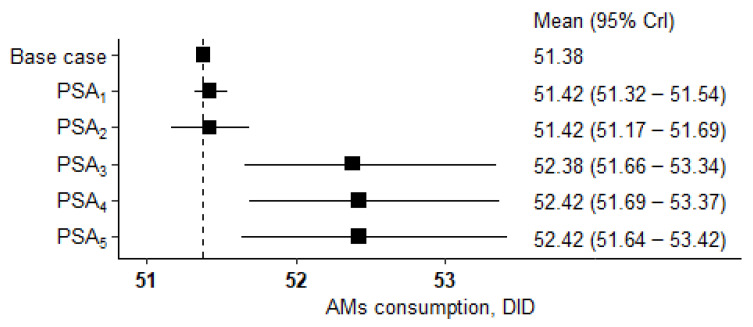
Forest plot of the results of PSAs to assess the robustness of the cDDD calculations depending on the input parameters’ variability.

The results of the probabilistic sensitivity analyses (PSAs) conducted to assess the robustness of the cDDD calculations depending on the variability in the input AMs (doses) and patients’ (weight and BSA) parameters are presented in Table 1 and Figure 1.

The analyses showed relatively low levels of variability in the cDDD values when there was reasonable variation in the input parameters. The maximal variability did not exceed 2% and was mainly related to changes in the doses (1.9% for separate dose variations), while the variability depending on the patients’ characteristics did not exceed 0.1%.

### 2.2. Comparative Evaluation of the Proposed Methodology and the Conventional One vs. the Objective Levels of AMC in Children 12 Years Old and Younger

Using the proposed methodology vs. the standard ATC/DDD methodology vs. objective levels based on PDDs, a comparative assessment of AMC levels in children aged 12 years and younger was performed at the pediatric surgery department of a multi-field hospital. A total of 1708.8 g of systemic AMs were consumed by the patients aged 1 month to 12 years during a 1-year period (4205 bed days).

**Figure 2:**
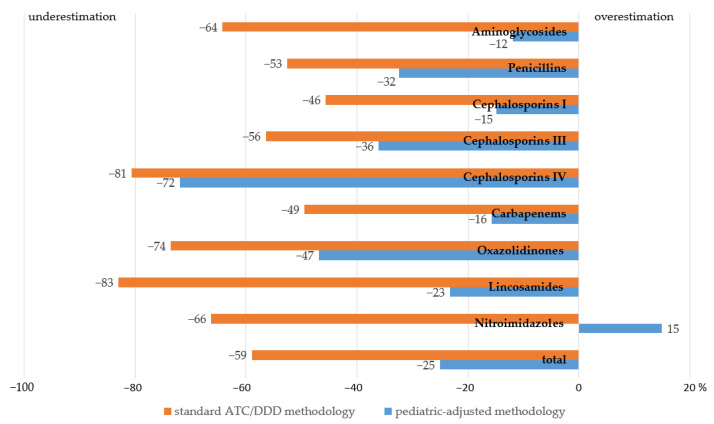
Deviations in AMC levels in the pediatric surgery department assessed by means of the proposed methodology and the standard ATC/DDD methodology compared to the objective levels based on PDDs, %.

The overall level of AMC consumption in the department was 18.9 DID according to the conventional methodology vs. 34.5 DID according to the proposed methodology, in comparison to 46 DID calculated by the PDDs extracted from the clinical records (Table 2). Thus, the assessment of the consumption using the standard methodology resulted in a 59% underestimation, whereas the proposed methodology had much lower levels of underestimation (25%) (Figure 2).

Underestimation by the standard methodology was the most significant for lincosamides (83%), fourth-generation cephalosporins (81%), oxazolidinones (74%), nitroimidazoles (66%), and aminoglycosides (64%); for the proposed methodology, it was most significant for fourth-generation cephalosporins (72%) and oxazolidinones (47%), whereas consumption of nitroimidazoles was overestimated by 15%. Altogether, the assessment of AMC by means of the proposed methodology turned out to be significantly more accurate compared to the standard ATC/DDD method.

### 2.3. Assessment of Variances in AMC in Multi-Field Hospitals with Pediatric Inpatients Calculated by Means of the Proposed Methodology vs. the Conventional One

The differences between AMC levels in multi-field hospitals with pediatric inpatients calculated by means of the conventional method vs. the proposed methodology are presented in the Supplementary Tables S5–S19. Overall, the use of the conventional ATC/DDD methodology led to the underestimation of AMC not only in a population of children of 12 years and younger but also in mixed-age populations, including adults. For a predefined set of AMs, representing the real practice of utilization in 3 multi-field hospitals combined, underestimation levels reached 321% in the base case scenario and 244% in the alternative one (Supplementary Tables S5 and S6).

**Figure 3:**
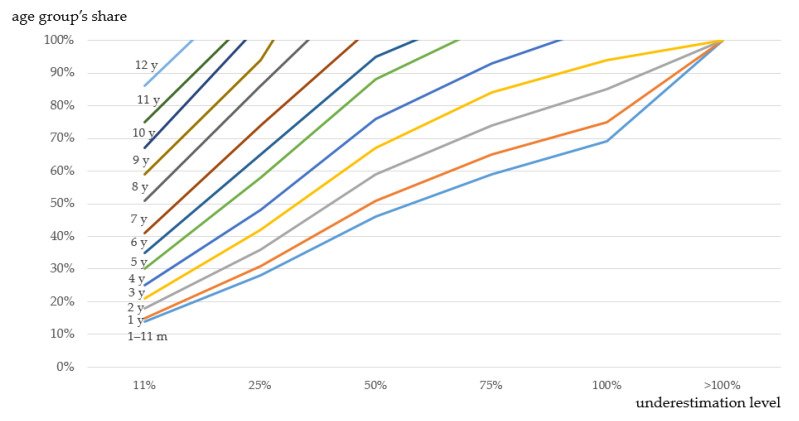
Level of underestimation of AMC in a multi-field hospital assessed by means of the standard ATC/DDD methodology in comparison to the pediatric-adjusted methodology for different age groups.

In the base case scenario, underestimation levels up to 10% and 11–24% were observed for all age groups in the case of the independent presence of pediatric inpatients of a single age in an adult population, with shares of children of 13% (1–11 months old) to 85% (12 years old) and 14–27% (1–11 months old) to ≥86% (12 years old) respectively. Moreover, a 25–49% underestimation of consumption was observed for the pediatric inpatients aged 1 month to 9 years for the shares of children from 28–45% (1–11 months old) to ≥94% (9 years old); a 50–74% underestimation was observed for the pediatric inpatients aged 1 month to 6 years for the shares of children from 46–68% (1–11 months old) to ≥95% (6 years old); 75–99% underestimation was observed for the pediatric inpatients aged 1 month to 4 years for the shares of children from 59–68% (1–11 months old) to ≥93% (4 years old); underestimation exceeded 100% for the pediatric inpatients aged 1 month to 3 years for the shares of children from ≥69% (1–11 months old) to ≥94% (3 years old) (Figure 3).

**Figure 4:**
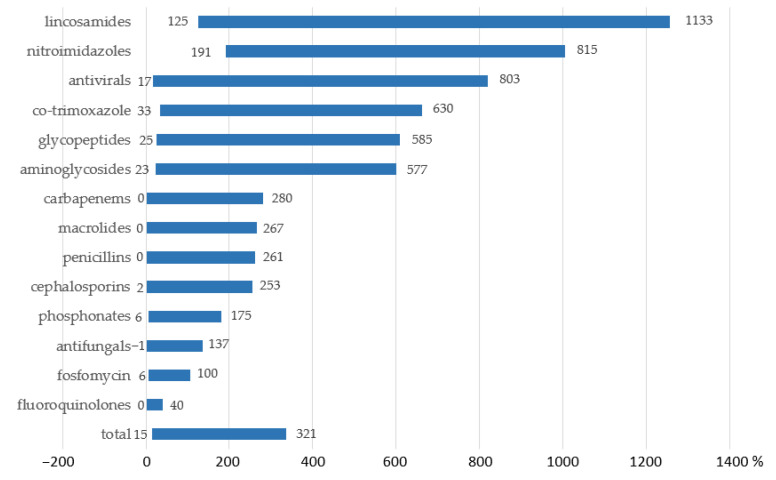
Differences in the levels of AMC in multi-field hospitals for different classes of drugs assessed by the conventional methodology vs. the proposed methodology (range for a virtual population, min for 100% of patients aged 12 years, max for 100% of patients aged 1–11 months), %.

Consumption rates of lincosamides (underestimation levels varied from 125% for 100% of patients aged 12 years to 1133% for 100% of patients aged 1–11 months), nitroimidazoles (191–815%), antiviral agents (17–803%), co-trimoxazole (33–630%), glycopeptides (25–585%), and aminoglycosides (23–577%) were most sensitive to age differences; this is probably due to significant age-dependent dose variations (Figure 4).

## 3. Discussion

Infections caused by AM-resistant bacteria are one of the major treatment challenges at present [1,2,3]. Amid increasing antibiotic resistance, with the development of new antibiotics being a difficult endeavor, the responsible use of existing drugs should be adhered to in every healthcare institution [15]. Reliable information on the qualitative and quantitative aspects of AMC is crucial for antimicrobial stewardship program interventions and for the control of effectiveness [16,17]. At the same time, AMC levels in hospitals can vary widely not only due to differences in institutions’ properties (size, specialization, inpatient population, etc.), local standards, and established practices of medical care, but also due to assessment approaches. Errors resulting from methodological issues can be substantial and significantly affect the interpretation of results.

Due to the demand for a reliable methodology, in 1981, the WHO proposed the ATC/DDD system as an international standard of drug consumption assessment. The main advantages of the methodology are its universal classification and dosing approaches, ease of use, the ability to input aggregate data without screening individual patients’ records, and its applicability for comparative assessments between healthcare institutions and benchmarking [7].

Despite its internationally recognized utility, the ATC/DDD methodology has a number of shortcomings related to the classification system, as well as the consumption measurement unit, DDD. The defined daily dose, or DDD, was developed and implemented by Norwegian researchers in collaboration with the Nordic Council on Medicines (Uppsala, Sweden) in 1976; it constitutes a universal unit for quantifying drug consumption, regardless of the specific drug, its cost, drug forms, or dosing units [7]. As an average maintenance dose per day for a drug used for its main indication in adults, the DDD is nearly always a compromise and only allows for a rough estimate of consumption. Thus, in a study performed by A. Muller et al. [18] in 2001 at a university hospital, a 40% overestimation of AMC levels was shown due to the deviations of DDDs from the prescribed doses. A similar trend was demonstrated in a number of other studies [8,19].

The problem becomes exponentially worse in patients with special dosing approaches, such as critically ill patients, patients with renal or hepatic impairment, and children. Adult DDDs do not correlate with actual doses in children, particularly those younger than six years, which can lead to the underestimation of drug consumption in this age group and in a total population of inpatients. The differences in the main indications for some AMs and the widespread off-label use of drugs in pediatric practice [20,21] may cause additional differences in dosing approaches.

Over the past two decades, alternative measures of antibiotic consumption have been proposed, including the direct measurement of the number of days of therapy (DOT), which is recommended by the American Centers for Disease Control and Prevention (CDC) [22], or prescribed daily doses (PDD). However, both approaches are time consuming and labor intensive and require the collection of information from the individual patients’ records, while the specific metric to accurately quantify pediatric AM use based on the aggregate data remains unresolved.

The main obstacles in the development of a universal assessment method to estimate drug consumption in children include the wide range of doses used, which necessitates an individual approach to each age period, as well as the unpredictability of the age structure of a pediatric population in a hospital during any given period of time. Body weight being the main determinant factor of dosing in children can present another problem, as the information about patient demographics and individual drug prescriptions is often limited in real practice, leading to age being used as a proxy indicator for body weight.

For instance, in 2005, Z. C. Zhang et al. [10] stratified patients by age to assess the adequacy of AM dosing in a pediatric population. Patients were divided into 5 groups, each with its own DDD values: 1–6 months, DDD_0_ = 1/7DDD; 6–12 months, DDD_1_ = 1/5DDD; 1–4 years, DDD_2_ = 1/3DDD; 4–8 years old, DDD_3_ = 1/2DDD; 8–15 years old, DDD_4_ = 2/3DDD. It should be noted that the developed DDDs were not specific to any drugs, were based on age without reference to body weight, and were not presented by the authors as a tool for consumption assessments.

The development of drug-specific cDDDs was performed by Lu Han et al. [11] in 2009. By cDDD, the researchers meant the estimated average maintenance dose of the drug per day per unit of weight used for the main indication in a child, similarly to standard DDDs, although, for a number of drugs, the cDDDs were presented as intervals.

In 2010, T. V. Liem et al. [9] proposed specific DDDs for the top 10 AMs used in neonates in intensive care units. The calculation of DDDs was carried out according to the estimated body weight of a 2 kg child, depending on the dosing standards and expert opinions. In 2011, on the basis of the studies performed by Z. C. Zhang and Lu Han, L. Zhang et al. [12] proposed an approach to the creation of a system of cDDDs to assess the optimal dosing of drugs in pediatric practice. It should be emphasized that all the abovementioned suggestions were presented solely for dosing assessment in the pediatric population of inpatients. Doses were not validated for the most part, and their application in the calculation of drug consumption has not been investigated.

In 2012, in an international pilot study performed in four European hospitals (in Great Britain, Greece, and Italy), A. Porta et al. [13] proposed an algorithm for the comparative assessment of AMC in pediatric patients. Doses of the most commonly prescribed drugs were stratified for 3 weight groups (body weight <10 kg, 10–25 kg, and ≥25 kg), with separate assessments of consumption levels for each group based on the standard ATC/DDD methodology. In 2014, R. Raastad et al. [14] compared the consumption of AMs in the pediatric population in dynamic terms, using specifically developed recommended daily doses per 100 kg days (RDDs/kg days) based on the national guidelines for pediatric antibiotic use, the length of stay, and the estimated weight for sex and age per national growth references. According to the authors, weight-adjusted assessments provided higher accuracy, particularly for neonates.

Despite the above-described efforts, the assessment of drug consumption in children is still far from perfect. In this article, the first attempt to propose an accurate and universal approach to AMC assessment in pediatric inpatients is described. The proposed methodology is based on the ATC/DDD system with the dose correction of adult DDDs in order to decrease discrepancies and to bring them closer to the real values for patients aged 12 years and younger, since the doses in older children generally correspond to those in the adults [23,24].

Just as in the abovementioned studies, for utility reasons, we elected not to incorporate body weight into the final formulas in favor of the easily performed stratification by age. In fact, the aggregated data on drug purchases and information on the age structure of patients and a total number of bed days over a given period of time are sufficient to estimate consumption with this method. Thus, it can be used in hospitals that are not equipped with the computerized pharmacy records of individual patients and do not routinely collect patients’ demographic characteristics and individual prescriptions.

cDDDs were calculated for each age group and were close to real clinical practice approaches per national summaries of product characteristics, with expert dose verification in the event of dose divergence. PSAs, which were performed to assess the robustness of calculations depending on the AM doses and the patients’ weight, as well as BSA variability, proved the stability of the cDDD values in the case of reasonable variation in the input parameters (≤2% variability).

The age-tailored cDDDs provided more accurate consumption assessments in comparison to the conventional ATC/DDD system (34.5 DID vs. 18.9 DID, respectively, vs. the PDD-based 46 DID). It is worth noting that, for some classes of drugs, such as lincosamides, fourth-generation cephalosporins, oxazolidinones, nitroimidazoles, and aminoglycosides, more pronounced underestimation by the standard methodology was observed. The divergence between the levels of consumption estimated by means of the proposed methodology and those based on the PDDs are probably related to differences between the recommended and the prescribed doses [8,25], the diversity of the recommended doses for different indications, and the common off-label dosing of AMs in children in real clinical practice [20,21].

Unaccounted underestimation errors in drug utilization monitoring can be expected not only in pediatric health facilities but also in multi-field hospitals with pediatric inpatients. The proposed methodology proved to be useful for AMC assessments in mixed-age populations, including adults. Thus, on a virtual population of inpatients for a predefined set of AMs, representing the real practice of utilization in 3 multi-field hospitals combined, the underestimation of consumption proved to be sizable (≥50%), even in institutions with moderate share of pediatric inpatients of early age groups (Supplementary Tables S5 and S6). Predictably, the consumption rates of some classes of drugs (e.g., lincosamides, nitroimidazoles, antiviral agents, co-trimoxazole, glycopeptides, and aminoglycosides) were more prone to be underestimated.

These considerations warrant the use of the proposed methodology not only in pediatric institutions but also in hospitals with a substantial share of pediatric inpatients. The methodology can be used in complex with the conventional one and retains the majority of the latter’s benefits, including the possibility of comparative assessments of consumption levels in the same health facility over time or among several health facilities with different age structures of inpatients.

At the same time, the proposed methodology has a number of limitations related to the approaches used to calculate cDDDs, as well as the variability in the source parameters (AM doses, body weight, and BSA values). AM doses based of the official prescription information or selected per experts’ opinion do not necessarily reflect the recommended or prescribed daily doses. The average body weight and BSA values provided by the WHO may not necessarily correctly represent these parameters in the patients of any given hospital around the world. Moreover, even presuming that the calculations of cDDDs were based on the objective input parameters, the real PDDs can still deviate from the cDDDs; this was the case in our study, where the levels of consumption estimated by means of the proposed methodology deviated from the real practice, although to a much smaller extent than those assessed using the conventional ATC/DDD methodology. The inability to provide cDDD values for infants due to registration shortages and the common off-label use of AMs in this age group can be considered another limitation of the proposed methodology.

Taking into account the abovementioned limitations, the proposed methodology still provides more accurate consumption estimates than the conventional method, both for a total set of AMs and for individual classes of drugs; it can be considered for the quantification of AM utilization in pediatric institutions and multi-field hospitals with a substantial share of pediatric inpatients.

Further research is needed to optimize children’s DDDs in relation to the prescribed ones and to study the usefulness of the proposed methodology in assessing AMC in healthcare facilities on a global level.

## 4. Materials and Methods

### 4.1. Basic Concept and Validation of the Proposed Pediatric-Adjusted Methodology

The proposed methodology is based on the conventional ATC/DDD methodology (WHO Collaborating Centre for Drug Statistics Methodology, Norwegian Institute of Public Health, Oslo, Norway) [7]. The adjusted children’s DDDs (cDDDs) were calculated in patients aged from 1 month to 12 years for the AMs of the following ATC groups: J01—antibacterials for systemic use, J02—antimycotics for systemic use, J05—antivirals for systemic use, and A07AA—antibiotics used as intestinal anti-infectives. The cDDD is presumed to be an average maintenance dose per day for a drug used for its main indication in a child with normal liver and kidney function.

The age subgroups were defined by year: 1–11 months, and then from 1 to 12 years. Doses for neonates were not proposed due to the scarcity of AM registrations and recommended doses for this age group. Dosage adjustment for children older than 12 years was considered unnecessary, since AM dosing regimens in that population are commonly similar to those in adults [23,24].

For each AM and considering the route of administration, the dose per growth unit was extracted from the national summaries of product characteristics (SmPC) [26]. If the dosing range was provided in the document, the dose was chosen based on the experts’ opinions (clinical pharmacologists, pediatricians, and infectious disease specialists who routinely administer AMs to children in clinical practice), with a tendency to use moderate-to-higher doses to correspond the current resistance progression [27,28]. The following standard dosing approaches were considered according to SmPCs (Supplementary Table S20):

1. Based on the child’s body weight (dose per kg),
2. Based on the child’s BSA (dose per m^2^),
3. Based on the child’s age.

Weight-for-age values (25th percentile, median, 75th percentile) were calculated based on the WHO’s child growth standards tables [29,30] as means for the corresponding values in boys and girls. For patients under 1 year of age, values were calculated as means of the 1–11-month weight data. For patients 11 to 12 years old, weight values were calculated based on the height and body mass index (BMI) data [31,32]:

$$weight\;\left(kg\right)=height\;{\left(m\right)}^{2}\times BMI,$$

BSA values were calculated using the R. D. Mosteller formula [33]:

$$BSA\;\left(m^{2}\right)=\sqrt{\frac{height\;\left(cm\right)\times weight\;\left(kg\right)}{3600}}.$$

Percentiles were converted to means and standard deviation (SD) values using the following formulas [34]:

$$mean=\frac{Q1+median+Q3}{3};\;SD=\frac{Q3-Q1}{1.35}.$$

The proposed estimation of AMC levels was performed as follows:

1. Calculate the weighted mean cDDD of an AM for a population of patients 1 month to 12 years old over the given period of time by multiplying the weight of each age subgroup in the population by the cDDD value for this age subgroup and summing the results:
  Weighted mean cDDD=cDDD1∗x1+cDDD2∗x2+…+cDDDn∗xn,
  where cDDD_n_ is the cDDD value for a given age group, and x_n_ is the share of this age subgroup in the population.
2. Divide the total amount of AM used in grams over the given period of time by the weighted mean cDDD for this agent to estimate the number of cDDDs used.
3. Calculate the level of AMC using the standard equation:
  $$AMC\;level=\frac{n\mathrm{cDDD}\;\prime \;100}{nb-d}$$
  where *n*_cDDD_ is the number of weighted mean cDDDs used over a given period of time, and *n*_b-d_ is the number of bed days for a population of patients aged 1 month to 12 years over a given period of time.

The result is expressed as the number of cDDDs per 100 bed days and reflects the percentage of patients aged 1 month to 12 years in a hospital who were treated daily with a given drug over a given time period. In the case of a mixed-age population of inpatients (1 month–12 years and older than 12 years, including adults), consumption was calculated separately for both age groups for a proportional share of AMs by means of the proposed and the conventional ATC/DDD methodologies.

The online AMRCalc (https://calc.antibiotic.ru/, accessed on 30 June 2023) tool, a purpose-built application based on the proposed methodology, was used to assess AMC in comparison to the conventional ATC/DDD methodology. Consumption data were expressed as DDDs per 100 bed days (DID). Here and elsewhere in the article, for visual convenience, we use the DID abbreviation for the expression of consumption levels measured by different units (DDD, cDDD, or PDD) as necessary.

For the validation of the proposed methodology, an analysis based on real clinical data, as well as several probabilistic sensitivity analyses (PSA), was conducted to assess the robustness of results depending on the input parameters’ (AM doses, weight, and BSA) variability. The clinical data for the calculations, such as the AM utilization data, the number of bed days, and the age structure of the patients, were extracted from the receipt notes of 3 typical multi-field hospitals from different regions of Russia for 2021 (1 pediatric, 2 mixed contingent). The analysis was conducted for the combined data to decrease fluctuating variations between the institutions.

Monte-Carlo simulations with 10,000 iterations were performed with the model inputs drawn randomly at each iteration from specific distributions assigned to each parameter. A uniform distribution was used to vary the dosage values in the allowed ranges according to the SmPCs. For the patient characteristics (weight, BSA), the normal distribution was used as recommended [35]. To assess the uncertainty, the 95% credible intervals (CrI) for Monte-Carlo simulations were also calculated.

### 4.2. Comparative Evaluation of the Proposed Methodology and the Conventional One vs. the Objective Levels of AMC in Children Aged 12 Years and Younger

A comparative evaluation of the proposed methodology in children 12 years of age and younger was performed by the assessment of AMC levels in a pediatric surgery department of a multi-field hospital during a 1-year period by means of the proposed methodology vs. the standard ATC/DDD methodology vs. objective levels based on PDDs. AM usage data, the number of bed-days, and the ages of the inpatients were extracted from the individual medical records of all patients aged 1 month to 12 years

### 4.3. Assessment of Variances in AMC in Multi-Field Hospitals with Pediatric Inpatients Calculated by Means of the Proposed Methodology vs. the Conventional One

To estimate differences in AMC in multi-field hospitals with pediatric inpatients calculated by means of the proposed methodology vs. the conventional one, a series of analyses was performed for a whole set of AMs, as well as the top classes used, on the combined data of the three aforementioned multi-field hospitals. The same dataset was used for a virtual cohort of inpatients with the pediatric share increasing by 1% for the separate age groups of 1–11 months, 1 year, 2 years, 3 years, 4 years, 5 years, 6 years, 7 years, 8 years, 9 years, 10 years, 11 years, and 12 years old; meanwhile, the rest of the patients were determined to be adults. AMs that are prohibited from use in children of any of the above age groups were excluded from the calculations for those age groups (base case) or set as having been used by adult patients (alternative case).

All statistical analyses were performed using R (version 4.3.0) and RStudio (version 2023.03.1+446) software.

## 5. Conclusions

Despite repeated attempts, the problem of developing a uniform and accurate approach to the assessment of AM consumption in children is still not resolved. In this article, we proposed a new uniform methodology for AMC assessment in pediatric inpatients based on the conventional ATC/DDD system. The proposed methodology was proven to provide more accurate quantitative estimations of AM utilization levels in patients aged 1 month to 12 years compared to the standard methodology, and it can be considered for AMC assessment in pediatric institutions and multi-field hospitals with a substantial share of pediatric inpatients.

## Figures and Tables

**Table 1:** Results of PSAs to assess the robustness of cDDD calculations depending on the input parameters’ variability, DID.

PSA #	PSA Description	Mean (SD)	95% CrI *	Difference from the Main Result (51.38 DID), %
1	Fixed AM doses. Weights and BSA values are randomly assigned for each AM in the structure.	51.42 (0.05)	51.32–51.54	0.1
2	Fixed AM doses. Weights, and BSA values are randomly assigned once to be the same for all AMs in the structure.	51.42 (0.13)	51.17–51.69	0.1
3	Doses are randomly assigned for each AM. Fixed mean weight and BSA values.	52.38 (0.50)	51.66–53.34	1.9
4	Doses are randomly assigned for each AM. Weight and BSA values are randomly assigned for each AM in the structure.	52.42 (0.49)	51.69–53.37	2
5	Doses are randomly assigned for each AM. Weight and BSA values are randomly assigned once to be the same for all AMs in the structure.	52.42 (0.51)	51.64–53.42	2

* CrI—credible interval.

**Table 2:** AMC levels in the pediatric surgery department assessed by means of the proposed methodology and the conventional ATC/DDD methodology in comparison with the objective levels based on PDDs.

AM Class	Consumption, g	Consumption, DID		
		ATC/DDD Methodology	Proposed Methodology	Based on PDDs
Aminoglycosides	73.8	2.42	5.97	6.78
Penicillins	27.9	0.37	0.53	0.78
Cephalosporins I	9.6	0.08	0.12	0.14
Cephalosporins III	1377.7	12.95	19.01	29.74
Cephalosporins IV	6.9	0.04	0.06	0.21
Carbapenems	69.2	0.78	1.31	1.55
Oxazolidinones	5.3	0.11	0.21	0.40
Lincosamides	2.7	0.04	0.16	0.21
Nitroimidazoles	135.7	2.08	7.11	6.18
Total	1708.8	18.9	34.5	46

## Data Availability

The data presented in this study are available from the corresponding author on reasonable request.

## Supplementary Materials

The following supporting information can be downloaded at: https://www.mdpi.com/article/10.3390/antibiotics12071162/s1, Table S1: Calculated cDDDs of AMs for children aged 1 month to 12 years; Table S2: Calculated weight values for children aged 1 month–12 years; Table S3: Calculated body surface area values for children aged 1 month–12 years; Table S4: AMs consumption assessment by means of the proposed methodology vs the conventional ATC/DDD methodology in a three multi-field hospitals (combined data); Table S5: Differences between AMs’ consumption levels calculated by means of novel pediatric-adjusted methodology vs conventional ATC/DDD methodology for a whole set of AMs (base case); Table S6: Differences between AMs’ consumption levels calculated by means of novel pediatric-adjusted methodology vs conventional ATC/DDD methodology for a whole set of AMs (alternative case); Table S7: Differences between AMs’ consumption levels calculated by means of novel pediatric-adjusted methodology vs conventional ATC/DDD methodology for aminoglycosides (base case); Table S8: Differences between AMs’ consumption levels calculated by means of novel pediatric-adjusted methodology vs conventional ATC/DDD methodology for penicillins (base case); Table S9: Differences between AMs’ consumption levels calculated by means of novel pediatric-adjusted methodology vs conventional ATC/DDD methodology for cephalosporins (base case); Table S10: Differences between AMs’ consumption levels calculated by means of novel pediatric-adjusted methodology vs conventional ATC/DDD methodology for carbapenems (base case); Table S11: Differences between AMs’ consumption levels calculated by means of novel pediatric-adjusted methodology vs conventional ATC/DDD methodology for fluoroquinolones (base case); Table S12: Differences between AMs’ consumption levels calculated by means of novel pediatric-adjusted methodology vs conventional ATC/DDD methodology for macrolides (base case); Table S13: Differences between AMs’ consumption levels calculated by means of novel pediatric-adjusted methodology vs conventional ATC/DDD methodology for lincosamides (base case); Table S14: Differences between AMs’ consumption levels calculated by means of novel pediatric-adjusted methodology vs conventional ATC/DDD methodology for glycopeptides (base case); Table S15: Differences between AMs’ consumption levels calculated by means of novel pediatric-adjusted methodology vs conventional ATC/DDD methodology for co-trimoxazole (base case); Table S16: Differences between AMs’ consumption levels calculated by means of novel pediatric-adjusted methodology vs conventional ATC/DDD methodology for nitroimidazoles (base case); Table S17: Differences between AMs’ consumption levels calculated by means of novel pediatric-adjusted methodology vs conventional ATC/DDD methodology for fosfomycin (base case); Table S18: Differences between AMs’ consumption levels calculated by means of novel pediatric-adjusted methodology vs conventional ATC/DDD methodology for antifungals (base case); Table S19: Differences between AMs’ consumption levels calculated by means of novel pediatric-adjusted methodology vs conventional ATC/DDD methodology for antivirals (base case); Table S20: Doses and dosing approaches from the national summaries of product characteristics used to calculate cDDDs of AMs for children aged 1 month to 12 years. (References [26,36] are cited in Supplementary Materials).
