# Supplementary material for: Assessment of Antimicrobial Consumption in Multi-Field Hospitals with Pediatric Inpatients: Conventional vs. Novel Pediatric-Adjusted Methodologies
Source: Antibiotics (Basel). 2023 Jul 7;12(7):1162. doi: 10.3390/antibiotics12071162 (PMC10376229; doi:10.3390/antibiotics12071162)
Supplement: Supplementary file 1 [file antibiotics-12-01162-s001.zip › antibiotics-2468906-supplementary.pdf]

SUPPLEMENTARY MATERIALS

Table of Contents

Supplementary Table S1. Calculated cDDDs of AMs for children aged 1 month to 12 years 2

Supplementary Table S2. Calculated weight values for children aged 1 month - 12 years 7

Supplementary Table S3. Calculated body surface area values for children aged 1 month - 12 years 7

Supplementary Table S4. AMs consumption assessment by means of the proposed methodology vs the conventional ATC/DDD methodology in a three multi-field hospitals (combined data) 8

Supplementary Table S5. Differences between AMs' consumption levels calculated by means of novel pediatric-adjusted methodology vs conventional ATC/DDD methodology for a whole set of AMs (base case)................ 10

Supplementary Table S6. Differences between AMs' consumption levels calculated by means of novel pediatric-adjusted methodology vs conventional ATC/DDD methodology for a whole set of AMs (alternative case)....... 12

Supplementary Table S7. Differences between AMs' consumption levels calculated by means of novel pediatric-adjusted methodology vs conventional ATC/DDD methodology for aminoglycosides (base case) 14

Supplementary Table S8. Differences between AMs' consumption levels calculated by means of novel pediatric-adjusted methodology vs conventional ATC/DDD methodology for penicillins (base case).............................. 16

Supplementary Table S9. Differences between AMs' consumption levels calculated by means of novel pediatric-adjusted methodology vs conventional ATC/DDD methodology for cephalosporins (base case) 18

Supplementary Table S10. Differences between AMs' consumption levels calculated by means of novel pediatric-adjusted methodology vs conventional ATC/DDD methodology for carbapenems (base case) 20

Supplementary Table S11. Differences between AMs' consumption levels calculated by means of novel pediatric-adjusted methodology vs conventional ATC/DDD methodology for fluoroquinolones (base case)................... 22

Supplementary Table S12. Differences between AMs' consumption levels calculated by means of novel pediatric-adjusted methodology vs conventional ATC/DDD methodology for macrolides (base case)............................. 24

Supplementary Table S13. Differences between AMs' consumption levels calculated by means of novel pediatric-adjusted methodology vs conventional ATC/DDD methodology for lincosamides (base case) 26

Supplementary Table S14. Differences between AMs' consumption levels calculated by means of novel pediatric-adjusted methodology vs conventional ATC/DDD methodology for glycopeptides (base case)......................... 28

Supplementary Table S15. Differences between AMs' consumption levels calculated by means of novel pediatric-adjusted methodology vs conventional ATC/DDD methodology for co-trimoxazole (base case) 30

Supplementary Table S16. Differences between AMs' consumption levels calculated by means of novel pediatric-adjusted methodology vs conventional ATC/DDD methodology for nitroimidazoles (base case) 32

Supplementary Table S17. Differences between AMs' consumption levels calculated by means of novel pediatric-adjusted methodology vs conventional ATC/DDD methodology for fosfomycin (base case) 34

Supplementary Table S18. Differences between AMs' consumption levels calculated by means of novel pediatric-adjusted methodology vs conventional ATC/DDD methodology for antifungals (base case) 36

Supplementary Table S19. Differences between AMs' consumption levels calculated by means of novel pediatric-adjusted methodology vs conventional ATC/DDD methodology for antivirals (base case) 38

Supplementary Table S20. Doses and dosing approaches from the national summaries of product characteristics used to calculate cDDDs of AMs for children aged 1 month to 12 years 40

Reference 44

Supplementary Table S1. Calculated cDDDs of AMs for children aged 1 month to 12 years, g

INN	Adm.R*	ATC code	1-11 months	1 year	2 years	3 years	4 years	5 years	6 years	7 years	8 years	9 years	10 years	11 years	12 years	Adult DDD [1]
Azithromycin	O	J01FA10	0.073	0.093	0.119	0.141	0.162	0.183	0.204	0.227	0.252	0.282	0.300	0.300	0.300	0.3
Azithromycin	P	J01FA10	0	0	0	0	0	0	0	0	0	0	0	0	0	0.5
Aztreonam	P	J01DF01	0.876	1.116	1.428	1.692	1.944	2.196	2.448	2.724	3.024	3.384	3.792	4.000	4.000	4
Amikacin	P	J01GB06	0.146	0.186	0.238	0.282	0.324	0.366	0.408	0.454	0.504	0.564	0.632	0.692	0.801	1
Amoxicillin	O	J01CA04	0.438	0.558	0.714	0.846	0.972	1.098	1.224	1.362	1.500	1.500	1.500	1.500	1.500	1.5
Amoxicillin and beta-lactamase inhibitor	O	J01CR02	0.438	0.558	0.714	0.846	0.972	1.098	1.224	1.362	1.500	1.500	1.500	1.500	1.500	1.5
Amoxicillin and beta-lactamase inhibitor	P	J01CR02	0.730	0.930	1.190	1.410	1.620	1.830	2.040	2.270	2.520	2.820	3.000	3.000	3.000	3
Ampicillin	P	J01CA01	1.460	1.860	2.380	2.820	3.240	3.660	4.080	4.540	5.040	5.640	6.000	6.000	6.000	6
Ampicillin and beta-lactamase inhibitor	P	J01CR01	1.460	1.860	2.380	2.820	3.240	3.660	4.080	4.540	5.040	5.640	6.000	6.000	6.000	6
Amphotericin B	P	J02AA01	0.011	0.014	0.018	0.021	0.024	0.027	0.031	0.034	0.035	0.035	0.035	0.035	0.035	0.035
Anidulafungin	P	J02AX06	0.011	0.014	0.018	0.021	0.024	0.027	0.031	0.034	0.038	0.042	0.047	0.053	0.060	0.1
Acyclovir	O	J05AB01	0	0	0	1.128	1.296	1.464	1.632	1.816	2.016	2.256	2.528	2.831	3.202	4
Acyclovir	P	J05AB01	0.438	0.558	0.714	0.846	0.972	1.098	1.224	1.362	1.512	1.692	1.896	2.123	2.204	4
Benzathine Benzylpenicillin	P	J01CE08	1.800	1.800	1.800	1.800	1.800	1.800	1.800	1.800	1.800	1.800	3.600	3.600	3.600	3.6
Benzylpenicillin	P	J01CE01	1.800	1.800	1.800	1.800	1.800	1.800	1.800	1.800	3.600	3.600	3.600	3.600	3.600	3.6
Bicillin-5	P	J01CE30	1.100	1.100	1.100	1.100	1.100	1.100	1.100	1.100	2.200	2.200	2.200	2.200	2.200	3.6
Valaciclivir	O	J05AB11	0	0	0	0	0	0	0	0	0	0	0	0	0	3
Valganciclovir	O	J05AB14	0	0	0	0	0	0	0	0	0	0	0	0	0	0.9
Vancomycin	O	A07AA09	0.292	0.372	0.476	0.564	0.648	0.732	0.816	0.908	1.008	1.128	1.264	1.416	1.601	2
Vancomycin	P	J01XA01	0.292	0.372	0.476	0.564	0.648	0.732	0.816	0.908	1.008	1.128	1.264	1.416	1.601	2
Voriconazole	O	J02AC03	0	0	0.190	0.226	0.259	0.293	0.326	0.363	0.403	0.451	0.506	0.566	0.640	0.4
Voriconazole	P	J02AC03	0	0	0.214	0.254	0.292	0.329	0.367	0.409	0.454	0.508	0.569	0.637	0.721	0.4
Ganciclovir	O	J05AB06	0	0	0	0	0	0	0	0	0	0	0	0	0	3
Ganciclovir	P	J05AB06	0	0	0	0	0	0	0	0	0	0	0	0	0	0.5
Gentamicin	P	J01GB03	0.037	0.056	0.071	0.071	0.081	0.092	0.102	0.114	0.126	0.141	0.158	0.177	0.200	0.24
Griseofulvin	O	D01BA01	0	0	0	0.141	0.162	0.183	0.204	0.227	0.252	0.282	0.316	0.354	0.400	0.5

Dalbavantsin	P	J01XA04	0	0	0	0	0	0	0	0	0	0	0	0	0	1.5
Daptomycin	P	J01XX09	0	0.093	0.107	0.127	0.146	0.165	0.184	0.159	0.176	0.197	0.221	0.248	0.200	0.28
Josamycin	O	J01FA07	0.365	0.465	0.595	0.705	0.810	0.915	1.020	1.135	1.260	1.410	1.580	1.770	2.000	2
Doxycycline	O	J01AA02	0	0	0	0	0	0	0	0	0.100	0.100	0.100	0.100	0.100	0.1
Doxycycline	P	J01AA02	0	0	0	0	0	0	0	0	0.100	0.100	0.100	0.100	0.100	0.1
Doripenem	P	J01DH04	0	0	0	0	0	0	0	0	0	0	0	0	0	1.5
Zanamivir	O	J05AH01	0	0	0	0	0	0.020	0.020	0.020	0.020	0.020	0.020	0.020	0.020	0.02
Isavuconazole	O	J02AC05	0	0	0	0	0	0	0	0	0	0	0	0	0	0.2
Isavuconazole	P	J02AC05	0	0	0	0	0	0	0	0	0	0	0	0	0	0.2
Imipenem and cilastatin	P	J01DH51	0.438	0.558	0.714	0.846	0.972	1.098	1.224	1.362	1.512	1.692	1.896	2.000	2.000	2
Itraconazole	O	J02AC02	0.037	0.047	0.060	0.071	0.081	0.092	0.102	0.114	0.126	0.141	0.158	0.177	0.200	0.2
Kanamycin	P	J01GB04	0.110	0.140	0.179	0.212	0.243	0.275	0.306	0.341	0.378	0.423	0.474	0.531	0.600	1
Caspofungin	P	J02AX04	0.026	0.031	0.037	0.043	0.048	0.052	0.057	0.061	0.070	0.070	0.070	0.070	0.070	0.05
Ketoconazole	O	J02AB02	0	0	0	0.100	0.100	0.100	0.100	0.100	0.100	0.100	0.200	0.200	0.200	0.2
Clarithromycin	O	J01FA09	0.110	0.140	0.179	0.212	0.243	0.275	0.306	0.341	0.378	0.423	0.474	0.500	0.500	0.5
Clarithromycin	P	J01FA09	0	0	0	0	0	0	0	0	0	0	0	0	0	1
Clindamycin	O	J01FF01	0	0	0	0	0	0	0	0	0	0	0	0	1.200	1.2
Clindamycin	P	J01FF01	0	0	0	0.564	0.648	0.732	0.816	0.908	1.008	1.128	1.264	1.416	1.601	1.8
Co-Trimoxazole	O	J01EE01	0.263	0.335	0.428	0.508	0.583	0.659	0.734	0.817	0.907	1.015	1.138	1.274	1.441	1.92
Co-Trimoxazole	P	J01EE01	0	0	0	0.508	0.583	0.659	0.734	0.817	0.907	1.015	1.138	1.274	1.441	1.92
Levofloxacin	O	J01MA12	0	0	0	0	0	0	0	0	0	0	0	0	0	0.5
Levofloxacin	P	J01MA12	0	0	0	0	0	0	0	0	0	0	0	0	0	0.5
Linezolid	O	J01XX08	0.219	0.279	0.357	0.423	0.486	0.549	0.612	0.681	0.756	0.846	0.948	1.062	1.200	1.2
Linezolid	P	J01XX08	0.219	0.279	0.357	0.423	0.486	0.549	0.612	0.681	0.756	0.846	0.948	1.062	1.200	1.2
Lincomycin	O	J01FF02	0	0	0	0.846	0.972	1.098	1.224	1.362	1.512	1.692	1.800	1.800	1.800	1.8
Lincomycin	P	J01FF02	0.146	0.186	0.238	0.282	0.324	0.366	0.408	0.454	0.504	0.564	0.632	0.708	0.801	1.8
Lomefloxacin	O	J01MA07	0	0	0	0	0	0	0	0	0	0	0	0	0	0.4
Meropenem	P	J01DH02	0.876	1.116	1.428	1.692	1.944	2.196	2.448	2.724	3.000	3.000	3.000	3.000	3.000	3
Metronidazole	O	P01AB01	0	0	0	0	0	0	0.500	0.500	0.500	0.500	0.500	0.500	0.500	2
Metronidazole	P	J01XD01	0.164	0.209	0.268	0.317	0.365	0.412	0.459	0.511	0.567	0.635	0.711	0.796	0.901	1.5
Midecamycin	O	J01FA03	0.365	0.465	0.595	0.705	0.810	0.915	1.020	1.135	1.200	1.200	1.200	1.200	1.200	1.2
Micafungin	P	J02AX05	0.029	0.037	0.048	0.056	0.065	0.073	0.082	0.091	0.100	0.100	0.100	0.100	0.100	0.1
minocycline	O	J01AA08	0	0	0	0	0	0	0	0	0.101	0.113	0.126	0.142	0.160	0.2
Moxifloxacin	O	J01MA14	0	0	0	0	0	0	0	0	0	0	0	0	0	0.4
Moxifloxacin	P	J01MA14	0	0	0	0	0	0	0	0	0	0	0	0	0	0.4
Natamycin	O	A07AA03	0	0	0	0.200	0.200	0.200	0.200	0.200	0.200	0.200	0.200	0.200	0.200	0.3

Netilmicin	P	J01GB07	0.055	0.070	0.089	0.106	0.122	0.137	0.153	0.170	0.189	0.212	0.237	0.265	0.300	0.35
Nimorazole	O	P01AB06	0	0	0	0.282	0.324	0.366	0.408	0.454	0.504	0.564	0.632	0.708	0.801	2
Nystatin	O	A07AA02	0	0	0	1.000	1.000	1.000	1.000	1.000	1.000	1.000	1.000	1.000	1.000	1.5
Nitroxoline	O	J01XX07	0	0	0	0.200	0.200	0.400	0.400	0.400	0.400	0.400	0.400	0.400	0.400	1
Nitrofurantoin	O	J01XE01	0	0	0	0	0	0	0.061	0.068	0.076	0.085	0.095	0.106	0.120	0.2
Nifuratel	O	G01AX05	0	0	0	0	0	0	0	0	0	0	0	0	0	0.6
Nifuroxazide	O	A07AX03	0.300	0.300	0.300	0.600	0.600	0.600	0.600	0.600	0.600	0.600	0.600	0.600	0.600	0.6
Norfloxacin	O	J01MA06	0	0	0	0	0	0	0	0	0	0	0	0	0	0.8
Oxacillin	P	J01CF04	1.000	1.000	2.000	2.000	2.000	2.000	2.000	2.000	2.000	2.000	2.000	2.000	2.000	2
Ornidazole	O	P01AB03	0	0	0	0.564	0.648	0.732	0.816	0.908	1.008	1.128	1.264	1.384	1.500	1.5
Ornidazole	P	J01XD03	0.219	0.279	0.357	0.423	0.486	0.549	0.612	0.681	0.756	0.846	0.948	1.000	1.000	1
Oseltamivir	O	J05AH02	0	0.060	0.060	0.060	0.090	0.090	0.090	0.090	0.120	0.120	0.120	0.120	0.120	0.15
Ofloxacin	O	J01MA01	0	0	0	0	0	0	0	0	0	0	0	0	0	0.4
Ofloxacin	P	J01MA01	0	0	0	0	0	0	0	0	0	0	0	0	0	0.4
Pefloxacin	O	J01MA03	0	0	0	0	0	0	0	0	0	0	0	0	0	0.8
Pefloxacin	P	J01MA03	0	0	0	0	0	0	0	0	0	0	0	0	0	0.8
Pipemidic acid	O	J01MB04	0	0	0	0	0	0	0	0	0	0	0	0	0	0.8
Piperacillin and beta-lactamase inhibitor	P	J01CR05	0	0	3.570	4.230	4.860	5.490	6.120	6.810	7.560	8.460	9.480	10.617	12.009	14
Posaconazole	O	J02AC04	0	0	0	0	0	0	0	0	0	0	0	0	0	0.3
Polymyxin B	P	J01XB02	0.018	0.023	0.030	0.035	0.041	0.046	0.051	0.057	0.063	0.071	0.079	0.088	0.100	0.15
Rimantadine	O	J05AC02	0	0.040	0.040	0.060	0.060	0.060	0.060	0.100	0.100	0.100	0.100	0.150	0.150	0.2
Rifaximin	O	A07AA11	0	0	0	0	0	0	0	0	0	0	0	0	0.600	0.6
Roxithromycin	O	J01FA06	0	0	0	0.113	0.130	0.146	0.163	0.182	0.202	0.226	0.253	0.283	0.300	0.3
Secnidazole	O	P01AB07	0	0	0	0.423	0.486	0.549	0.612	0.681	0.756	0.846	0.948	1.062	1.201	2
Sparfloxacin	O	J01MA09	0	0	0	0	0	0	0	0	0	0	0	0	0	0.2
Spectinomycin	P	J01XX04	0	0	0	0.564	0.648	0.732	0.816	0.908	1.008	1.128	1.264	1.416	1.601	3
Spiramycin	O	J01FA02	0	0	0	0	0	0	1.836	2.043	2.268	2.538	2.800	2.800	2.800	3
Streptomycin	P	J01GA01	0.146	0.186	0.238	0.282	0.324	0.366	0.408	0.454	0.504	0.564	0.632	0.708	0.801	1
Sultamicillin	O	J01CR04	0.365	0.465	0.595	0.705	0.750	0.750	0.750	0.750	0.750	0.750	0.750	0.750	0.750	1.5
Sulfadimethoxine	O	J01ED01	0.091	0.116	0.149	0.176	0.203	0.229	0.255	0.284	0.315	0.353	0.395	0.442	0.500	0.5
Sulfadimidine	O	J01EB03	0	0	0	2.115	2.430	2.745	3.060	3.405	3.780	4.000	4.000	4.000	4.000	4
Tedizolid	O	J01XX11	0	0	0	0	0	0	0	0	0	0	0	0	0	0.2
Tedizolid	P	J01XX11	0	0	0	0	0	0	0	0	0	0	0	0	0	0.2
Teicoplanin	P	J01XA02	0.073	0.093	0.119	0.141	0.162	0.183	0.204	0.227	0.252	0.282	0.316	0.354	0.400	0.4
Terbinafine	O	D01BA02	0	0	0	0.125	0.125	0.125	0.125	0.125	0.125	0.125	0.125	0.125	0.125	0.25

Tetracycline	O	J01AA07	0	0	0	0	0	0	0	0	1.000	1.000	1.000	1.000	1.000	1
Thiamphenicol	P	J01BA02	0.250	0.250	0.250	0.500	0.500	0.500	0.500	0.750	0.750	0.750	0.750	0.750	0.750	1.5
Tigecycline	P	J01AA12	0	0	0	0	0	0	0	0	0.060	0.068	0.076	0.085	0.096	0.1
Tinidazole	O	P01AB02	0	0	0	0	0	0	0	0	0	0	0	0	2.000	2
Umifenovir	O	J05AX13	0	0	0.200	0.200	0.200	0.200	0.400	0.400	0.400	0.400	0.400	0.400	0.800	0.8
Favipiravir	O	J05AX27	0	0	0	0	0	0	0	0	0	0	0	0	0	1.6
Famciclovir	O	J05AB09	0	0	0	0	0	0	0	0	0	0	0	0	0	0.75
Fluconazole	O	J02AC01	0	0	0	0.169	0.194	0.200	0.200	0.200	0.200	0.200	0.200	0.200	0.200	0.2
Fluconazole	P	J02AC01	0.088	0.112	0.143	0.169	0.194	0.200	0.200	0.200	0.200	0.200	0.200	0.200	0.200	0.2
Fosfomycin	O	J01XX01	0	0	0	0	0	2.000	2.000	2.000	2.000	2.000	2.000	2.000	2.000	3
Fosfomycin	P	J01XX01	2.920	3.720	4.760	5.640	6.480	7.320	8.000	8.000	8.000	8.000	8.000	8.000	8.000	8
Fusidic acid	O	J01XC01	0	0	0	0.846	0.972	0.732	0.816	0.908	1.008	1.128	1.264	1.416	1.500	1.5
Furazidin	O	J01XE03	0	0	0	0	0	0	0	0	0	0	0	0	0	0.3
Furazolidone	O	G01AX06	0	0	0	0.141	0.162	0.183	0.204	0.227	0.252	0.282	0.316	0.354	0.400	0.6
Chloramphenicol	O	J01BA01	0	0	0	0.705	0.810	0.915	1.020	1.135	1.260	1.410	1.580	1.770	2.002	3
Cefazolin	P	J01DB04	0.730	0.930	1.190	1.410	1.620	1.830	2.040	2.270	2.520	2.820	3.000	3.000	3.000	3
Cefalexin	O	J01DB01	0.730	0.930	1.190	1.410	1.620	1.830	2.000	2.000	2.000	2.000	2.000	2.000	2.000	2
Cefamandol	P	J01DC03	1.095	1.395	1.785	2.115	2.430	2.745	3.060	3.405	3.780	4.230	4.740	5.309	6.000	6
Cefditoren	O	J01DD16	0	0	0	0	0	0	0	0	0	0	0	0	0.400	0.4
cefepime	P	J01DE01	1.095	1.395	1.785	2.115	2.430	2.745	3.060	3.405	3.780	4.000	4.000	4.000	4.000	4
Cefixime	O	J01DD08	0.058	0.074	0.095	0.113	0.130	0.146	0.163	0.182	0.202	0.226	0.253	0.283	0.320	0.4
Cefoxitin	P	J01DC01	1.168	1.488	1.904	2.256	2.592	2.928	3.264	3.632	4.032	4.512	5.056	5.662	6.000	6
Cefoperazone	P	J01DD12	1.460	1.860	2.380	2.820	3.240	3.660	4.000	4.000	4.000	4.000	4.000	4.000	4.000	4
Cefoperazone and beta-lactamase inhibitor	P	J01DD62	0.584	0.744	0.952	1.128	1.296	1.464	1.632	1.816	2.016	2.256	2.528	2.831	3.202	4
Cefotaxime	P	J01DD01	0.730	0.930	1.190	1.410	1.620	1.830	2.040	2.270	2.520	2.820	3.160	3.539	4.000	4
Cefpir	P	J01DE02	0	0	0	0	0	0	0	0	0	0	0	0	4.000	4
Ceftazidime	P	J01DD02	1.095	1.395	1.785	2.115	2.430	2.745	3.060	3.405	3.780	4.000	4.000	4.000	4.000	4
Ceftazidime and beta-lactamase inhibitor	P	J01DD52	0	0	0	0	0	0	0	0	0	0	0	0	0	6
Ceftaroline	P	J01DI02	0.175	0.223	0.428	0.508	0.583	0.659	0.734	0.817	0.907	1.015	1.138	1.200	1.200	1.2
Ceftolosan and beta-lactamase inhibitor	P	J01DI54	0	0	0	0	0	0	0	0	0	0	0	0	0	3
Ceftriaxone	P	J01DD04	0.730	0.930	1.190	1.410	1.620	1.830	2.000	2.000	2.000	2.000	2.000	2.000	2.000	2

Cefuroxime	P	J01DC02	0.730	0.930	1.190	1.410	1.620	1.830	2.040	2.270	2.520	2.820	3.000	3.000	3.000	3
Cefuroxime	O	J01DC03	0.110	0.140	0.179	0.212	0.243	0.275	0.306	0.341	0.378	0.423	0.474	0.500	0.500	0.5
Ciprofloxacin	O	J01MA02	0	0	0	0	0	0.732	0.816	0.908	1.000	1.000	1.000	1.000	1.000	1
Ciprofloxacin	P	J01MA02	0	0	0	0	0	0.549	0.612	0.681	0.756	0.800	0.800	0.800	0.800	0.8
Erythromycin	O	J01FA01	0	0	0	0	0	0	0	0	0	0	0	0	0	1
Erythromycin	P	J01FA01	0.365	0.465	0.595	0.705	0.810	0.915	1.000	1.000	1.000	1.000	1.000	1.000	1.000	1
Ertapenem	P	J01DH03	0.219	0.279	0.357	0.423	0.486	0.549	0.612	0.681	0.756	0.846	0.948	1.000	1.000	1

Adm.R - rout of administration, O – oral, P – parenteral

Supplementary Table S2. Calculated weight values for children aged 1 month - 12 years, kg [2]

Age, years	Q1	Me	Q3	M	SD
1-11 month	6.7	7.3	7.9	7.3	0.89
1 year	8.6	9.3	10.0	9.3	1.07
2 years	11.0	11.8	12.8	11.9	1.37
3 years	13.0	14.1	15.4	14.1	1.78
4 years	14.8	16.2	17.8	16.3	2.15
5 years	16.6	18.2	20.2	18.3	2.63
6 years	18.6	20.4	22.4	20.5	2.89
7 years	20.6	22.6	25.0	22.8	3.33
8 years	22.8	25.2	28.0	25.3	3.89
9 years	25.4	28.2	31.5	28.3	4.56
10 years	28.2	31.6	35.4	31.7	5.30
11 years	30.9	35.4	41.2	35.8	7.59
12 years	34.8	40.0	46.6	40.5	8.78

Q1 - 1st quartile (25th percentile); Me - median (50th percentile); Q3 - 3rd quartile (75th percentile); M – mean; SD - standard deviation

Supplementary Table S3. Calculated body surface area values for children aged 1 month - 12 years, kg [2]

Age, years	Q1	Me	Q3	M	SD
1 year	0.35	0.37	0.38	0.37	0.028
2 years	0.42	0.44	0.46	0.44	0.032
3 years	0.51	0.53	0.56	0.53	0.041
4 years	0.58	0.61	0.65	0.61	0.050
5 years	0.64	0.68	0.72	0.68	0.059
6 years	0.70	0.75	0.80	0.75	0.069
7 years	0.76	0.81	0.86	0.81	0.075
8 years	0.82	0.87	0.93	0.88	0.083
9 years	0.88	0.94	1.01	0.95	0.094
10 years	0.95	1.02	1.09	1.02	0.105
11 years	1.02	1.10	1.18	1.10	0.118
12 years	1.09	1.19	1.30	1.20	0.155
1 year	1.19	1.29	1.42	1.30	0.171

Q1 - 1st quartile (25th percentile); Me - median (50th percentile); Q3 - 3rd quartile (75th percentile); M – mean; SD - standard deviation

Supplementary Table S4. AMs consumption assessment by means of the proposed methodology vs the conventional ATC/DDD methodology in a three multi-field hospitals (combined data)

INN	Adm.R*	ATC code	AMs consumption		
			g	proposed methodology, DID	ATC/DDD methodology, DID
Azithromycin	O	J01FA10	4619.6	3.32	3.06
Azithromycin	P	J01FA10	933.5	0.37	0.37
Amikacin	P	J01GB06	3125	0.70	0.62
Amoxicillin	O	J01CA04	4689.5	0.66	0.62
Amoxicillin and beta-lactamase inhibitor	O	J01CR02	23336.6	3.30	3.09
Amoxicillin and beta-lactamase inhibitor	P	J01CR02	21788	1.56	1.44
Ampicillin	P	J01CA01	700	0.03	0.02
Ampicillin and beta-lactamase inhibitor	P	J01CR01	1085.5	0.04	0.04
Acyclovir	O	J05AB01	1816	0.1	0.09
Acyclovir	P	J05AB01	618.25	0.04	0.03
Benzathine Benzylpenicillin	P	J01CE08	42	0.003	0.002
Valaciclivir	O	J05AB11	125	0.01	0.01
Valganciclovir	O	J05AB14	27	0.01	0.01
Vancomycin	P	J01XA01	7274.9	0.82	0.72
Voriconazole	O	J02AC03	11.2	0.01	0.01
Voriconazole	P	J02AC03	114.4	0.06	0.06
Ganciclovir	P	J05AB06	9.5	0.004	0.004
Gentamicin	P	J01GB03	561.6	0.52	0.46
Josamycin	O	J01FA07	25	0.003	0.002
Doxycycline	O	J01AA02	422	0.84	0.84
Doripenem	P	J01DH04	345.5	0.05	0.05
Zanamivir	O	J05AH01	0.2	0.002	0.002
Imipenem and cilastatin	P	J01DH51	2690	0.29	0.27
Caspofungin	P	J02AX04	7.6	0.03	0.03
Clarithromycin	O	J01FA09	1251.5	0.54	0.5
Clarithromycin	P	J01FA09	324	0.06	0.06
Clindamycin	P	J01FF01	9	0.001	0.001
Co-Trimoxazole	O	J01EE01	5350.56	0.63	0.55
Levofloxacin	O	J01MA12	4920	1.96	1.96
Levofloxacin	P	J01MA12	2742.5	1.09	1.09
Linezolid	O	J01XX08	335.4	0.06	0.06
Linezolid	P	J01XX08	960	0.18	0.16
Lincomycin	P	J01FF02	7701	0.99	0.85
Meropenem	P	J01DH02	23880	1.69	1.58
Metronidazole	O	P01AB01	12420	1.32	1.23
Metronidazole	P	J01XD01	8116.1	1.24	1.08
Midecamycin	O	J01FA03	535.2	0.09	0.09
Micafungin	P	J02AX05	20.45	0.04	0.04
Moxifloxacin	O	J01MA14	24	0.01	0.01
Moxifloxacin	P	J01MA14	223.6	0.11	0.11
Nifuroxazide	O	A07AX03	225.72	0.08	0.07
Norfloxacin	O	J01MA06	40	0.01	0.01
Oxacillin	P	J01CF04	465	0.05	0.05
Oseltamivir	O	J05AH02	118.2	0.17	0.16
Piperacillin and beta-lactamase inhibitor	P	J01CR05	2896	0.04	0.04
Posaconazole	O	J02AC04	63	0.04	0.04
Polymyxin B	P	J01XB02	297	0.45	0.39
Rimantadine	O	J05AC02	89	0.1	0.09

Tigecycline	P	J01AA12	2.5	0.005	0.005
Umifenovir	O	J05AX13	1238	0.33	0.31
Favipiravir	O	J05AX27	544	0.07	0.07
Fluconazole	O	J02AC01	580.8	0.58	0.58
Fluconazole	P	J02AC01	326.2	0.34	0.32
Fosfomycin	O	J01XX01	170	0.01	0.01
Fosfomycin	P	J01XX01	3720	0.10	0.09
Furazidin	O	J01XE03	4.5	0.003	0.003
Chloramphenicol	O	J01BA01	240	0.02	0.02
Cefazolin	P	J01DB04	16602	1.19	1.1
Cefepime	P	J01DE01	28693	1.53	1.43
Cefixime	O	J01DD08	38.4	0.02	0.02
Cefoperazone and beta-lactamase inhibitor	P	J01DD62	37666.5	2.12	1.87
Cefotaxime	P	J01DD01	34081	1.88	1.69
Ceftazidime	P	J01DD02	4552	0.24	0.23
Ceftazidime and beta-lactamase inhibitor	P	J01DD52	242	0.01	0.01
Ceftaroline	P	J01DI02	44.4	0.01	0.01
Ceftriaxone	P	J01DD04	134041.5	13.99	13.32
Cefuroxime	P	J01DC02	3372.75	0.02	0.02
Cefuroxime	O	J01DC03	50	0.24	0.22
Ciprofloxacin	O	J01MA02	15495	3.1	3.08
Ciprofloxacin	P	J01MA02	5363.6	1.35	1.33
Ertapenem	P	J01DH03	2539	0.55	0.5
**Total**				**51.38**	**48.3**

Adm.R - rout of administration. O – oral. P – parenteral

Influence of pediatric inpatients' share on the total level of AMs consumption in a multi-field hospital, %

Level of underestimation of AMs consumption assessed by means of the standard ATC/DDD methodology in comparison to the pediatric-adjusted methodology:

- ≤10%
- 11%-24%
- 25%-49%
- 50%-74%
- 75%-99%
- ≥100%

Supplementary Table S5. Differences between AMs' consumption levels calculated by means of novel pediatric-adjusted methodology vs conventional ATC/DDD methodology for a whole set of AMs (base case), %

Share / Age group	1-11 month	1 year	2 years	3 years	4 years	5 years	6 years	7 years	8 years	9 years	10 years	11 years	12 years
**1%**	1	1	1	0	0	0	0	0	0	0	0	0	0
**2%**	1	1	1	1	1	1	1	0	0	0	0	0	0
**3%**	2	2	2	1	1	1	1	1	0	0	0	0	0
**4%**	3	3	2	2	2	1	1	1	1	1	0	0	0
**5%**	4	3	3	2	2	2	1	1	1	1	1	0	0
**6%**	4	4	3	3	2	2	2	1	1	1	1	1	0
**7%**	5	5	4	3	3	2	2	1	1	1	1	1	0
**8%**	6	5	5	4	3	3	2	2	1	1	1	1	1
**9%**	7	6	5	4	4	3	2	2	1	1	1	1	1
**10%**	8	7	6	5	4	3	3	2	2	1	1	1	1
**11%**	9	8	6	5	4	4	3	2	2	1	1	1	1
**12%**	9	8	7	6	5	4	3	3	2	2	1	1	1
**13%**	10	9	8	6	5	4	3	3	2	2	1	1	1
**14%**	11	10	8	7	6	5	4	3	2	2	2	1	1
**15%**	12	11	9	8	6	5	4	3	3	2	2	1	1
**16%**	13	12	10	8	7	5	4	4	3	2	2	1	1
**17%**	14	12	10	9	7	6	5	4	3	2	2	2	1
**18%**	15	13	11	9	8	6	5	4	3	2	2	2	1
**19%**	16	14	12	10	8	6	5	4	3	3	2	2	1
**20%**	17	15	12	10	9	7	6	5	3	3	2	2	1
**21%**	18	16	13	11	9	7	6	5	4	3	2	2	2
**22%**	19	17	14	12	9	8	6	5	4	3	3	2	2
**23%**	20	18	15	12	10	8	6	5	4	3	3	2	2
**24%**	21	18	15	13	10	8	7	6	4	3	3	2	2
**25%**	22	19	16	13	11	9	7	6	4	4	3	2	2
**26%**	23	20	17	14	11	9	7	6	5	4	3	3	2
**27%**	24	21	18	15	12	10	8	6	5	4	3	3	2
**28%**	25	22	18	15	13	10	8	7	5	4	3	3	2
**29%**	26	23	19	16	13	10	8	7	5	4	3	3	2
**30%**	28	24	20	17	14	11	9	7	5	4	4	3	2
**31%**	29	25	21	17	14	11	9	8	6	5	4	3	2
**32%**	30	26	22	18	15	12	10	8	6	5	4	3	2
**33%**	31	27	23	19	15	12	10	8	6	5	4	3	3
**34%**	32	28	23	19	16	12	10	8	6	5	4	4	3
**35%**	34	29	24	20	16	13	11	9	7	5	4	4	3
**36%**	35	31	25	21	17	13	11	9	7	6	4	4	3
**37%**	36	32	26	21	18	14	11	9	7	6	5	4	3
**38%**	38	33	27	22	18	14	12	10	7	6	5	4	3
**39%**	39	34	28	23	19	15	12	10	8	6	5	4	3
**40%**	41	35	29	24	19	15	13	10	8	6	5	4	3
**41%**	42	37	30	24	20	16	13	11	8	6	5	4	3
**42%**	44	38	31	25	21	16	13	11	8	7	5	5	3
**43%**	45	39	32	26	21	17	14	11	9	7	6	5	4
**44%**	47	40	33	27	22	17	14	12	9	7	6	5	4
**45%**	48	42	34	28	22	18	15	12	9	7	6	5	4
**46%**	50	43	35	28	23	18	15	12	9	8	6	5	4
**47%**	52	44	36	29	24	19	15	13	10	8	6	5	4

**48%**	53	46	37	30	25	19	16	13	10	8	6	5	4
**49%**	55	47	38	31	25	20	16	13	10	8	7	6	4
**50%**	57	49	39	32	26	20	17	14	10	8	7	6	4
**51%**	59	50	41	33	27	21	17	14	11	9	7	6	4
**52%**	61	52	42	34	27	21	18	14	11	9	7	6	5
**53%**	62	53	43	35	28	22	18	15	11	9	7	6	5
**54%**	64	55	44	36	29	23	19	15	12	9	8	6	5
**55%**	67	57	46	37	30	23	19	16	12	10	8	7	5
**56%**	69	58	47	38	30	24	20	16	12	10	8	7	5
**57%**	71	60	48	39	31	24	20	16	13	10	8	7	5
**58%**	73	62	49	40	32	25	21	17	13	10	8	7	5
**59%**	75	64	51	41	33	26	21	17	13	11	9	7	6
**60%**	78	66	52	42	34	26	22	18	13	11	9	7	6
**61%**	80	67	54	43	35	27	22	18	14	11	9	8	6
**62%**	82	69	55	44	35	28	23	19	14	11	9	8	6
**63%**	85	71	57	45	36	28	23	19	15	12	10	8	6
**64%**	88	73	58	46	37	29	24	19	15	12	10	8	6
**65%**	90	76	60	48	38	30	25	20	15	12	10	8	6
**66%**	93	78	61	49	39	30	25	20	16	13	10	9	7
**67%**	96	80	63	50	40	31	26	21	16	13	11	9	7
**68%**	99	82	65	51	41	32	26	21	16	13	11	9	7
**69%**	102	85	66	53	42	32	27	22	17	14	11	9	7
**70%**	105	87	68	54	43	33	28	22	17	14	11	9	7
**71%**	108	90	70	55	44	34	28	23	18	14	12	10	7
**72%**	112	92	72	57	45	35	29	24	18	15	12	10	8
**73%**	115	95	74	58	46	36	30	24	18	15	12	10	8
**74%**	119	98	76	59	47	36	30	25	19	15	12	10	8
**75%**	123	100	78	61	49	37	31	25	19	16	13	11	8
**76%**	127	103	80	62	50	38	32	26	20	16	13	11	8
**77%**	131	106	82	64	51	39	33	26	20	16	13	11	9
**78%**	135	110	84	66	52	40	33	27	21	17	14	11	9
**79%**	139	113	86	67	53	41	34	28	21	17	14	12	9
**80%**	144	116	89	69	55	42	35	28	22	18	14	12	9
**81%**	149	120	91	71	56	43	36	29	22	18	15	12	9
**82%**	154	123	93	73	57	44	37	30	23	18	15	13	10
**83%**	159	127	96	74	59	45	37	30	23	19	15	13	10
**84%**	165	131	99	76	60	46	38	31	24	19	16	13	10
**85%**	170	135	101	78	62	47	39	32	24	20	16	13	10
**86%**	177	140	104	80	63	48	40	33	25	20	16	14	11
**87%**	183	144	107	83	65	49	41	33	26	21	17	14	11
**88%**	190	149	110	85	66	50	42	34	26	21	17	14	11
**89%**	197	154	114	87	68	51	43	35	27	22	18	15	11
**90%**	204	159	117	89	70	53	44	36	27	22	18	15	12
**91%**	213	164	121	92	72	54	45	37	28	23	19	16	12
**92%**	221	170	124	94	74	55	47	38	29	23	19	16	12
**93%**	230	176	128	97	75	57	48	38	30	24	19	16	13
**94%**	240	183	132	100	77	58	49	39	30	25	20	17	13
**95%**	251	190	136	103	80	59	50	40	31	25	20	17	13
**96%**	263	197	141	106	82	61	51	41	32	26	21	18	14
**97%**	275	205	146	109	84	63	53	42	33	27	22	18	14
**98%**	289	214	151	112	86	64	54	44	34	27	22	19	15
**99%**	304	223	156	116	89	66	56	45	34	28	23	19	15
**100%**	321	233	162	119	91	68	57	46	35	29	23	20	15

Supplementary Table S6. Differences between AMs' consumption levels calculated by means of novel pediatric-adjusted methodology vs conventional ATC/DDD methodology for a whole set of AMs (alternative case), %

Share / Age group	1-11 month	1 year	2 years	3 years	4 years	5 years	6 years	7 years	8 years	9 years	10 years	11 years	12 years
1%	1	0	0	0	0	0	0	0	0	0	0	0	0
2%	1	1	1	1	1	1	0	0	0	0	0	0	0
3%	2	2	1	1	1	1	1	1	0	0	0	0	0
4%	2	2	2	1	1	1	1	1	1	0	0	0	0
5%	3	3	2	2	2	1	1	1	1	1	0	0	0
6%	3	3	3	2	2	2	1	1	1	1	1	0	0
7%	4	4	3	3	2	2	2	1	1	1	1	1	0
8%	5	4	4	3	3	2	2	2	1	1	1	1	0
9%	5	5	4	3	3	3	2	2	1	1	1	1	1
10%	6	5	5	4	3	3	2	2	2	1	1	1	1
11%	6	6	5	4	4	3	3	2	2	1	1	1	1
12%	7	6	5	5	4	3	3	2	2	1	1	1	1
13%	8	7	6	5	4	4	3	3	2	2	1	1	1
14%	8	8	6	5	5	4	3	3	2	2	1	1	1
15%	9	8	7	6	5	4	4	3	2	2	2	1	1
16%	10	9	7	6	5	5	4	3	2	2	2	1	1
17%	11	9	8	7	6	5	4	3	3	2	2	1	1
18%	11	10	9	7	6	5	4	4	3	2	2	2	1
19%	12	11	9	8	6	6	5	4	3	2	2	2	1
20%	13	11	10	8	7	6	5	4	3	3	2	2	1
21%	13	12	10	9	7	6	5	4	3	3	2	2	1
22%	14	13	11	9	7	7	6	5	4	3	2	2	1
23%	15	13	11	10	8	7	6	5	4	3	2	2	2
24%	16	14	12	10	8	7	6	5	4	3	3	2	2
25%	17	15	12	11	9	8	6	5	4	3	3	2	2
26%	17	15	13	11	9	8	7	6	4	3	3	2	2
27%	18	16	14	11	9	8	7	6	4	4	3	2	2
28%	19	17	14	12	10	9	7	6	5	4	3	3	2
29%	20	18	15	12	10	9	8	6	5	4	3	3	2
30%	21	18	16	13	11	9	8	7	5	4	3	3	2
31%	22	19	16	14	11	10	8	7	5	4	3	3	2
32%	23	20	17	14	12	10	9	7	5	4	4	3	2
33%	24	21	17	15	12	11	9	7	6	5	4	3	2
34%	25	22	18	15	12	11	9	8	6	5	4	3	2
35%	26	23	19	16	13	11	10	8	6	5	4	3	3
36%	27	23	19	16	13	12	10	8	6	5	4	3	3
37%	28	24	20	17	14	12	10	8	6	5	4	4	3
38%	29	25	21	17	14	13	11	9	7	5	4	4	3
39%	30	26	22	18	15	13	11	9	7	6	5	4	3
40%	31	27	22	19	15	13	11	9	7	6	5	4	3
41%	32	28	23	19	16	14	12	10	7	6	5	4	3
42%	33	29	24	20	16	14	12	10	8	6	5	4	3
43%	34	30	25	20	17	15	12	10	8	6	5	4	3
44%	35	31	25	21	17	15	13	10	8	7	5	4	3
45%	37	32	26	22	18	16	13	11	8	7	5	5	3
46%	38	33	27	22	18	16	14	11	9	7	6	5	4
47%	39	34	28	23	19	16	14	11	9	7	6	5	4

**48%**	40	35	29	24	19	17	14	12	9	7	6	5	4
**49%**	42	36	30	24	20	17	15	12	9	8	6	5	4
**50%**	43	37	30	25	20	18	15	12	10	8	6	5	4
**51%**	45	38	31	26	21	18	16	13	10	8	6	5	4
**52%**	46	40	32	27	22	19	16	13	10	8	7	6	4
**53%**	47	41	33	27	22	19	16	13	10	8	7	6	4
**54%**	49	42	34	28	23	20	17	14	11	9	7	6	4
**55%**	50	43	35	29	23	20	17	14	11	9	7	6	5
**56%**	52	45	36	30	24	21	18	14	11	9	7	6	5
**57%**	54	46	37	30	25	21	18	15	12	9	8	6	5
**58%**	55	47	38	31	25	22	19	15	12	10	8	7	5
**59%**	57	49	39	32	26	22	19	16	12	10	8	7	5
**60%**	59	50	40	33	27	23	20	16	12	10	8	7	5
**61%**	61	52	41	34	27	24	20	16	13	10	8	7	5
**62%**	63	53	43	35	28	24	21	17	13	11	9	7	5
**63%**	64	55	44	36	29	25	21	17	13	11	9	7	6
**64%**	66	56	45	36	29	25	22	18	14	11	9	8	6
**65%**	69	58	46	37	30	26	22	18	14	11	9	8	6
**66%**	71	59	47	38	31	27	23	18	14	12	9	8	6
**67%**	73	61	49	39	32	27	23	19	15	12	10	8	6
**68%**	75	63	50	40	32	28	24	19	15	12	10	8	6
**69%**	77	65	51	41	33	29	24	20	15	13	10	9	7
**70%**	80	67	53	42	34	29	25	20	16	13	10	9	7
**71%**	82	68	54	43	35	30	26	21	16	13	11	9	7
**72%**	85	70	55	45	36	31	26	21	17	13	11	9	7
**73%**	88	72	57	46	36	31	27	22	17	14	11	9	7
**74%**	90	75	58	47	37	32	27	22	17	14	11	10	7
**75%**	93	77	60	48	38	33	28	23	18	14	12	10	8
**76%**	96	79	62	49	39	33	29	23	18	15	12	10	8
**77%**	99	81	63	50	40	34	29	24	19	15	12	10	8
**78%**	102	84	65	52	41	35	30	24	19	15	13	11	8
**79%**	106	86	67	53	42	36	31	25	20	16	13	11	8
**80%**	109	89	68	54	43	37	32	26	20	16	13	11	8
**81%**	113	92	70	56	44	38	32	26	20	17	13	11	9
**82%**	117	94	72	57	45	38	33	27	21	17	14	12	9
**83%**	121	97	74	59	46	39	34	27	21	17	14	12	9
**84%**	125	100	76	60	47	40	35	28	22	18	14	12	9
**85%**	129	103	78	62	49	41	36	29	22	18	15	12	10
**86%**	134	107	81	63	50	42	36	29	23	19	15	13	10
**87%**	139	110	83	65	51	43	37	30	24	19	16	13	10
**88%**	144	114	85	67	52	44	38	31	24	20	16	13	10
**89%**	149	118	88	68	54	45	39	32	25	20	16	14	11
**90%**	155	121	90	70	55	46	40	32	25	21	17	14	11
**91%**	161	126	93	72	56	47	41	33	26	21	17	14	11
**92%**	168	130	96	74	58	49	42	34	27	22	18	15	11
**93%**	175	135	99	76	59	50	43	35	27	22	18	15	12
**94%**	182	140	102	78	61	51	44	36	28	23	18	15	12
**95%**	190	145	105	81	63	52	45	37	29	23	19	16	12
**96%**	199	151	109	83	64	54	47	37	29	24	19	16	13
**97%**	209	157	112	86	66	55	48	38	30	24	20	17	13
**98%**	219	163	116	88	68	56	49	39	31	25	20	17	13
**99%**	231	170	120	91	70	58	50	40	32	26	21	18	14
**100%**	244	178	125	94	72	59	52	42	33	26	22	18	14

Supplementary Table S7. Differences between AMs' consumption levels calculated by means of novel pediatric-adjusted methodology vs conventional ATC/DDD methodology for aminoglycosides (base case), %

Share / Age group	1-11 month	1 year	2 years	3 years	4 years	5 years	6 years	7 years	8 years	9 years	10 years	11 years	12 years
1%	1	1	1	1	1	1	1	0	0	0	0	0	0
2%	2	2	2	1	1	1	1	1	1	1	1	1	0
3%	3	3	2	2	2	2	2	2	1	1	1	1	1
4%	3	3	3	3	3	3	2	2	2	2	1	1	1
5%	5	4	4	4	3	3	3	3	2	2	2	1	1
6%	5	5	5	5	4	4	4	3	3	3	2	2	1
7%	6	6	6	5	5	5	4	4	3	3	2	2	1
8%	7	7	6	6	6	5	5	5	4	3	3	2	1
9%	8	8	7	7	6	6	6	5	5	4	3	3	2
10%	9	9	8	8	7	7	6	6	5	4	4	3	2
11%	10	10	9	8	8	7	7	6	6	5	4	3	2
12%	11	11	10	9	9	8	8	7	6	5	5	3	2
13%	13	12	11	10	10	9	8	7	7	6	5	4	2
14%	14	13	12	11	10	10	9	8	7	6	5	4	3
15%	15	14	13	12	11	10	10	9	8	7	6	4	3
16%	16	15	14	13	12	11	10	9	8	7	6	5	3
17%	17	16	15	14	13	12	11	10	9	8	7	5	3
18%	18	17	16	15	14	13	12	11	10	8	7	5	3
19%	19	18	17	16	15	14	13	11	10	9	7	6	4
20%	21	19	18	17	15	14	13	12	11	9	8	6	4
21%	22	21	19	18	16	15	14	13	11	10	8	6	4
22%	23	22	20	19	17	16	15	13	12	10	9	7	4
23%	24	23	21	20	18	17	16	14	13	11	9	7	5
24%	26	24	22	21	19	18	16	15	13	11	9	7	5
25%	27	26	23	22	20	19	17	16	14	12	10	8	5
26%	28	27	25	23	21	20	18	16	14	13	10	8	5
27%	30	28	26	24	22	20	19	17	15	13	11	8	5
28%	31	29	27	25	23	21	20	18	16	14	11	8	6
29%	33	31	28	26	24	22	20	19	16	14	12	9	6
30%	34	32	29	27	25	23	21	19	17	15	12	9	6
31%	36	34	31	28	26	24	22	20	18	15	12	9	6
32%	37	35	32	30	27	25	23	21	18	16	13	10	6
33%	39	37	33	31	28	26	24	22	19	16	13	10	7
34%	41	38	35	32	29	27	25	22	20	17	14	10	7
35%	43	40	36	33	31	28	26	23	21	17	14	11	7
36%	44	41	38	35	32	29	27	24	21	18	15	11	7
37%	46	43	39	36	33	30	28	25	22	19	15	12	7
38%	48	45	41	37	34	31	29	26	23	19	16	12	8
39%	50	46	42	38	35	33	30	27	23	20	16	12	8
40%	52	48	44	40	37	34	31	28	24	21	17	13	8
41%	54	50	45	41	38	35	31	28	25	21	17	13	8
42%	56	52	47	43	39	36	33	29	26	22	18	13	8
43%	58	54	48	44	40	37	34	30	26	22	18	14	9
44%	60	56	50	46	42	38	35	31	27	23	19	14	9
45%	62	58	52	47	43	39	36	32	28	24	19	14	9
46%	65	60	54	49	45	41	37	33	29	24	20	15	9
47%	67	62	55	50	46	42	38	34	30	25	20	15	9

**48%**	69	64	57	52	47	43	39	35	30	26	21	15	10
**49%**	72	66	59	54	49	44	40	36	31	26	21	16	10
**50%**	74	68	61	56	50	46	41	37	32	27	22	16	10
**51%**	77	71	63	57	52	47	43	38	33	28	22	17	10
**52%**	80	73	65	59	53	49	44	39	34	29	23	17	11
**53%**	82	76	67	61	55	50	45	40	35	29	23	17	11
**54%**	85	78	69	63	57	51	46	41	36	30	24	18	11
**55%**	88	81	72	65	58	53	48	42	37	31	24	18	11
**56%**	91	83	74	67	60	54	49	43	37	31	25	19	12
**57%**	94	86	76	69	62	56	50	44	38	32	26	19	12
**58%**	98	89	79	71	64	57	51	46	39	33	26	19	12
**59%**	101	92	81	73	65	59	53	47	40	34	27	20	12
**60%**	105	95	84	75	67	61	54	48	41	34	27	20	13
**61%**	108	98	86	77	69	62	56	49	42	35	28	21	13
**62%**	112	101	89	79	71	64	57	50	43	36	28	21	13
**63%**	116	105	92	82	73	66	58	51	44	37	29	21	13
**64%**	120	108	95	84	75	67	60	53	45	38	30	22	13
**65%**	124	112	97	87	77	69	61	54	46	38	30	22	14
**66%**	129	116	100	89	79	71	63	55	47	39	31	23	14
**67%**	133	119	103	92	81	73	64	57	48	40	31	23	14
**68%**	138	123	107	94	84	75	66	58	50	41	32	23	14
**69%**	143	127	110	97	86	77	68	59	51	42	33	24	15
**70%**	148	132	113	100	88	79	69	61	52	43	33	24	15
**71%**	153	136	117	103	91	81	71	62	53	43	34	25	15
**72%**	159	141	121	106	93	83	73	63	54	44	35	25	15
**73%**	165	146	124	109	96	85	75	65	55	45	35	26	16
**74%**	171	151	128	112	98	87	76	66	56	46	36	26	16
**75%**	177	156	132	115	101	89	78	68	58	47	37	27	16
**76%**	184	161	136	119	104	92	80	70	59	48	37	27	16
**77%**	191	167	141	122	107	94	82	71	60	49	38	27	17
**78%**	198	173	145	126	110	96	84	73	61	50	39	28	17
**79%**	206	179	150	129	113	99	86	74	63	51	39	28	17
**80%**	214	185	155	133	116	101	88	76	64	52	40	29	17
**81%**	223	192	160	137	119	104	90	78	65	53	41	29	18
**82%**	232	199	165	141	122	107	92	79	67	54	41	30	18
**83%**	242	207	170	145	125	109	94	81	68	55	42	30	18
**84%**	252	215	176	150	129	112	97	83	69	56	43	31	19
**85%**	263	223	182	154	132	115	99	85	71	57	44	31	19
**86%**	274	232	188	159	136	118	101	87	72	58	44	32	19
**87%**	287	241	195	164	140	121	104	88	73	59	45	32	19
**88%**	300	251	201	169	144	124	106	90	75	60	46	33	20
**89%**	314	261	208	174	148	127	109	92	77	61	47	33	20
**90%**	329	272	216	180	152	130	111	94	78	62	47	34	20
**91%**	346	283	223	185	156	134	114	96	80	63	48	34	20
**92%**	363	296	232	191	161	137	117	99	81	65	49	35	21
**93%**	382	309	240	198	165	141	119	101	83	66	50	35	21
**94%**	403	323	249	204	170	145	122	103	84	67	51	36	21
**95%**	425	338	259	211	175	149	125	105	86	68	51	36	21
**96%**	450	354	269	218	180	152	128	107	88	69	52	37	22
**97%**	477	371	279	225	186	157	131	110	90	71	53	37	22
**98%**	507	390	291	233	192	161	135	112	91	72	54	38	22
**99%**	540	410	303	241	197	165	138	115	93	73	55	38	22
**100%**	577	433	315	250	203	170	141	117	95	74	56	39	23

Supplementary Table S8. Differences between AMs' consumption levels calculated by means of novel pediatric-adjusted methodology vs conventional ATC/DDD methodology for penicillins (base case), %

Share / Age group	1-11 month	1 year	2 years	3 years	4 years	5 years	6 years	7 years	8 years	9 years	10 years	11 years	12 years
1%	1	1	1	0	0	0	0	0	0	0	0	0	0
2%	2	1	1	1	1	1	0	0	0	0	0	0	0
3%	2	2	2	1	1	1	1	0	0	0	0	0	0
4%	3	3	2	2	2	1	1	1	0	0	0	0	0
5%	4	3	3	2	2	2	1	1	0	0	0	0	0
6%	5	4	3	3	2	2	1	1	0	0	0	0	0
7%	5	5	4	3	3	2	2	1	0	0	0	0	0
8%	6	5	5	4	3	3	2	1	0	0	0	0	0
9%	7	6	5	4	4	3	2	1	0	0	0	0	0
10%	8	7	6	5	4	3	2	1	1	0	0	0	0
11%	9	8	6	5	4	3	3	2	1	0	0	0	0
12%	9	8	7	6	5	4	3	2	1	0	0	0	0
13%	10	9	8	6	5	4	3	2	1	0	0	0	0
14%	11	10	8	7	6	5	3	2	1	0	0	0	0
15%	12	11	9	7	6	5	4	2	1	0	0	0	0
16%	13	12	10	8	7	5	4	2	1	0	0	0	0
17%	14	12	10	9	7	6	4	2	1	0	0	0	0
18%	15	13	11	9	7	6	4	3	1	0	0	0	0
19%	16	14	12	10	8	6	5	3	1	0	0	0	0
20%	17	15	12	10	8	7	5	3	1	0	0	0	0
21%	18	16	13	11	9	7	5	3	1	0	0	0	0
22%	19	17	14	11	9	7	5	3	1	0	0	0	0
23%	20	17	14	12	10	8	6	3	1	0	0	0	0
24%	21	18	15	12	10	8	6	4	1	1	0	0	0
25%	22	19	16	13	11	8	6	4	1	1	0	0	0
26%	23	20	17	14	11	9	6	4	1	1	0	0	0
27%	24	21	17	14	12	9	7	4	1	1	0	0	0
28%	25	22	18	15	12	9	7	4	1	1	0	0	0
29%	26	23	19	15	13	10	7	4	1	1	0	0	0
30%	28	24	20	16	13	10	7	4	2	1	0	0	0
31%	29	25	20	17	13	11	8	5	2	1	0	0	0
32%	30	26	21	17	14	11	8	5	2	1	0	0	0
33%	31	27	22	18	14	11	8	5	2	1	0	0	0
34%	32	28	23	19	15	12	8	5	2	1	0	0	0
35%	34	29	24	19	16	12	9	5	2	1	0	0	0
36%	35	30	25	20	16	12	9	5	2	1	0	0	0
37%	36	31	25	21	17	13	9	6	2	1	0	0	0
38%	38	33	26	21	17	13	9	6	2	1	0	0	0
39%	39	34	27	22	18	14	10	6	2	1	0	0	0
40%	41	35	28	23	18	14	10	6	2	1	0	0	0
41%	42	36	29	23	19	14	10	6	2	1	0	0	0
42%	43	37	30	24	19	15	11	6	2	1	0	0	0
43%	45	39	31	25	20	15	11	7	2	1	0	0	0
44%	46	40	32	26	20	16	11	7	2	1	0	0	0
45%	48	41	33	26	21	16	11	7	2	1	0	0	0
46%	50	42	34	27	21	16	12	7	2	1	0	0	0
47%	51	44	35	28	22	17	12	7	3	1	0	0	0

**48%**	53	45	36	29	23	17	12	7	3	1	0	0	0
**49%**	55	46	37	29	23	18	13	8	3	1	0	0	0
**50%**	56	48	38	30	24	18	13	8	3	1	0	0	0
**51%**	58	49	39	31	24	19	13	8	3	1	0	0	0
**52%**	60	51	40	32	25	19	14	8	3	1	0	0	0
**53%**	62	52	41	33	26	19	14	8	3	1	0	0	0
**54%**	64	54	42	33	26	20	14	8	3	1	0	0	0
**55%**	66	55	43	34	27	20	14	9	3	1	0	0	0
**56%**	68	57	44	35	27	21	15	9	3	1	0	0	0
**57%**	70	58	45	36	28	21	15	9	3	1	0	0	0
**58%**	72	60	47	37	29	22	15	9	3	1	0	0	0
**59%**	74	62	48	38	29	22	16	9	3	1	0	0	0
**60%**	76	63	49	39	30	23	16	9	3	1	0	0	0
**61%**	78	65	50	39	31	23	16	10	3	1	0	0	0
**62%**	81	67	52	40	31	24	17	10	3	1	0	0	0
**63%**	83	69	53	41	32	24	17	10	4	1	0	0	0
**64%**	86	71	54	42	33	25	17	10	4	1	0	0	0
**65%**	88	73	55	43	33	25	18	10	4	1	0	0	0
**66%**	91	75	57	44	34	26	18	11	4	1	0	0	0
**67%**	93	77	58	45	35	26	18	11	4	1	0	0	0
**68%**	96	79	60	46	36	27	19	11	4	2	0	0	0
**69%**	99	81	61	47	36	27	19	11	4	2	0	0	0
**70%**	102	83	62	48	37	28	19	11	4	2	0	0	0
**71%**	105	85	64	49	38	28	20	11	4	2	0	0	0
**72%**	108	87	65	50	39	29	20	12	4	2	0	0	0
**73%**	111	90	67	51	39	29	20	12	4	2	0	0	0
**74%**	114	92	69	52	40	30	21	12	4	2	0	0	0
**75%**	118	94	70	54	41	30	21	12	4	2	0	0	0
**76%**	121	97	72	55	42	31	21	12	4	2	0	0	0
**77%**	125	100	73	56	42	31	22	13	4	2	0	0	0
**78%**	129	102	75	57	43	32	22	13	4	2	0	0	0
**79%**	132	105	77	58	44	32	22	13	5	2	0	0	0
**80%**	137	108	79	59	45	33	23	13	5	2	0	0	0
**81%**	141	110	80	61	46	34	23	13	5	2	0	0	0
**82%**	145	113	82	62	46	34	23	14	5	2	0	0	0
**83%**	149	116	84	63	47	35	24	14	5	2	0	0	0
**84%**	154	119	86	64	48	35	24	14	5	2	0	0	0
**85%**	159	123	88	66	49	36	25	14	5	2	0	0	0
**86%**	164	126	90	67	50	36	25	14	5	2	0	0	0
**87%**	169	129	92	68	51	37	25	15	5	2	0	0	0
**88%**	174	133	94	70	52	38	26	15	5	2	0	0	0
**89%**	180	136	96	71	53	38	26	15	5	2	0	0	0
**90%**	186	140	98	72	54	39	26	15	5	2	0	0	0
**91%**	192	144	101	74	55	39	27	15	5	2	0	0	0
**92%**	198	148	103	75	55	40	27	16	5	2	0	0	0
**93%**	204	152	105	77	56	41	28	16	6	2	0	0	0
**94%**	211	156	108	78	57	41	28	16	6	2	0	0	0
**95%**	219	161	110	80	58	42	28	16	6	2	0	0	0
**96%**	226	165	113	81	59	43	29	16	6	2	0	0	0
**97%**	234	170	115	83	60	43	29	17	6	2	0	0	0
**98%**	243	175	118	85	61	44	30	17	6	2	0	0	0
**99%**	251	180	121	86	63	45	30	17	6	2	0	0	0
**100%**	261	185	123	88	64	45	30	17	6	2	0	0	0

Supplementary Table S9. Differences between AMs' consumption levels calculated by means of novel pediatric-adjusted methodology vs conventional ATC/DDD methodology for cephalosporins (base case), %

Share / Age group	1-11 month	1 year	2 years	3 years	4 years	5 years	6 years	7 years	8 years	9 years	10 years	11 years	12 years
1%	1	1	0	0	0	0	0	0	0	0	0	0	0
2%	1	1	1	1	1	0	0	0	0	0	0	0	0
3%	2	2	2	1	1	1	0	0	0	0	0	0	0
4%	3	2	2	2	1	1	1	0	0	0	0	0	0
5%	4	3	3	2	2	1	1	1	0	0	0	0	0
6%	4	4	3	2	2	1	1	1	1	0	0	0	0
7%	5	4	4	3	2	2	1	1	1	1	0	0	0
8%	6	5	4	3	3	2	1	1	1	1	0	0	0
9%	7	6	5	4	3	2	1	1	1	1	0	0	0
10%	7	6	5	4	3	2	1	1	1	1	1	0	0
11%	8	7	6	5	4	3	2	1	1	1	1	0	0
12%	9	8	6	5	4	3	2	2	1	1	1	0	0
13%	10	9	7	5	4	3	2	2	1	1	1	1	0
14%	11	9	7	6	5	3	2	2	1	1	1	1	0
15%	12	10	8	6	5	3	2	2	2	1	1	1	0
16%	12	11	9	7	5	4	2	2	2	1	1	1	0
17%	13	11	9	7	6	4	3	2	2	1	1	1	0
18%	14	12	10	8	6	4	3	2	2	1	1	1	0
19%	15	13	10	8	6	4	3	2	2	1	1	1	0
20%	16	14	11	9	7	5	3	3	2	2	1	1	0
21%	17	15	12	9	7	5	3	3	2	2	1	1	0
22%	18	15	12	10	7	5	3	3	2	2	1	1	0
23%	19	16	13	10	8	6	4	3	2	2	1	1	0
24%	20	17	14	11	8	6	4	3	3	2	1	1	0
25%	21	18	14	11	9	6	4	3	3	2	1	1	0
26%	22	19	15	12	9	6	4	3	3	2	2	1	1
27%	23	20	16	12	9	7	4	4	3	2	2	1	1
28%	24	20	16	13	10	7	5	4	3	2	2	1	1
29%	25	21	17	13	10	7	5	4	3	2	2	1	1
30%	26	22	18	14	11	7	5	4	3	2	2	1	1
31%	27	23	18	14	11	8	5	4	3	2	2	1	1
32%	28	24	19	15	11	8	5	4	3	3	2	1	1
33%	29	25	20	15	12	8	5	5	4	3	2	1	1
34%	31	26	20	16	12	9	6	5	4	3	2	1	1
35%	32	27	21	17	13	9	6	5	4	3	2	1	1
36%	33	28	22	17	13	9	6	5	4	3	2	1	1
37%	34	29	23	18	13	10	6	5	4	3	2	2	1
38%	36	30	24	18	14	10	6	5	4	3	2	2	1
39%	37	31	24	19	14	10	7	6	4	3	2	2	1
40%	38	32	25	20	15	10	7	6	4	3	2	2	1
41%	40	33	26	20	15	11	7	6	5	3	3	2	1
42%	41	34	27	21	16	11	7	6	5	4	3	2	1
43%	42	36	28	21	16	11	8	6	5	4	3	2	1
44%	44	37	28	22	17	12	8	6	5	4	3	2	1
45%	45	38	29	23	17	12	8	7	5	4	3	2	1
46%	47	39	30	23	18	12	8	7	5	4	3	2	1
47%	48	40	31	24	18	13	8	7	5	4	3	2	1

**48%**	50	42	32	25	19	13	9	7	6	4	3	2	1
**49%**	51	43	33	25	19	14	9	7	6	4	3	2	1
**50%**	53	44	34	26	20	14	9	8	6	4	3	2	1
**51%**	55	45	35	27	20	14	9	8	6	4	3	2	1
**52%**	56	47	36	27	21	15	10	8	6	5	3	2	1
**53%**	58	48	37	28	21	15	10	8	6	5	3	2	1
**54%**	60	49	38	29	22	15	10	8	7	5	3	2	1
**55%**	62	51	39	30	22	16	10	9	7	5	4	2	1
**56%**	64	52	40	30	23	16	11	9	7	5	4	2	1
**57%**	65	54	41	31	23	17	11	9	7	5	4	2	1
**58%**	67	55	42	32	24	17	11	9	7	5	4	3	1
**59%**	69	57	43	33	24	17	12	9	7	5	4	3	1
**60%**	71	58	44	33	25	18	12	10	7	5	4	3	1
**61%**	74	60	45	34	26	18	12	10	8	6	4	3	1
**62%**	76	62	46	35	26	19	12	10	8	6	4	3	1
**63%**	78	63	47	36	27	19	13	10	8	6	4	3	1
**64%**	80	65	49	37	27	19	13	11	8	6	4	3	1
**65%**	83	67	50	38	28	20	13	11	8	6	4	3	1
**66%**	85	69	51	38	29	20	14	11	9	6	4	3	1
**67%**	87	70	52	39	29	21	14	11	9	6	5	3	1
**68%**	90	72	53	40	30	21	14	11	9	6	5	3	1
**69%**	93	74	55	41	31	22	15	12	9	7	5	3	2
**70%**	95	76	56	42	31	22	15	12	9	7	5	3	2
**71%**	98	78	58	43	32	23	15	12	9	7	5	3	2
**72%**	101	80	59	44	33	23	16	13	10	7	5	3	2
**73%**	104	83	60	45	33	24	16	13	10	7	5	3	2
**74%**	107	85	62	46	34	24	16	13	10	7	5	3	2
**75%**	110	87	63	47	35	25	17	13	10	7	5	3	2
**76%**	113	89	65	48	36	25	17	14	10	8	5	4	2
**77%**	117	92	66	49	36	26	17	14	11	8	5	4	2
**78%**	120	94	68	50	37	26	18	14	11	8	6	4	2
**79%**	124	97	70	52	38	27	18	14	11	8	6	4	2
**80%**	128	99	71	53	39	27	18	15	11	8	6	4	2
**81%**	131	102	73	54	40	28	19	15	11	8	6	4	2
**82%**	136	105	75	55	40	28	19	15	12	8	6	4	2
**83%**	140	108	77	56	41	29	20	16	12	9	6	4	2
**84%**	144	111	78	58	42	30	20	16	12	9	6	4	2
**85%**	149	114	80	59	43	30	20	16	12	9	6	4	2
**86%**	153	117	82	60	44	31	21	17	13	9	6	4	2
**87%**	158	120	84	61	45	32	21	17	13	9	6	4	2
**88%**	164	124	86	63	46	32	22	17	13	9	7	4	2
**89%**	169	127	88	64	47	33	22	18	13	10	7	4	2
**90%**	175	131	91	66	48	34	23	18	13	10	7	4	2
**91%**	181	135	93	67	49	34	23	18	14	10	7	4	2
**92%**	187	139	95	69	50	35	24	19	14	10	7	4	2
**93%**	194	143	98	70	51	36	24	19	14	10	7	5	2
**94%**	201	147	100	72	52	36	25	19	15	10	7	5	2
**95%**	208	152	103	74	53	37	25	20	15	11	7	5	2
**96%**	216	157	106	75	54	38	26	20	15	11	7	5	2
**97%**	224	162	108	77	55	39	26	20	15	11	7	5	2
**98%**	233	167	111	79	57	40	27	21	16	11	8	5	2
**99%**	243	173	114	81	58	40	27	21	16	11	8	5	2
**100%**	253	179	118	83	59	41	28	22	16	11	8	5	2

Supplementary Table S10. Differences between AMs' consumption levels calculated by means of novel pediatric-adjusted methodology vs conventional ATC/DDD methodology for carbapenems (base case), %

Share / Age group	1-11 month	1 year	2 years	3 years	4 years	5 years	6 years	7 years	8 years	9 years	10 years	11 years	12 years
1%	1	1	1	0	0	0	0	0	0	0	0	0	0
2%	1	1	1	1	1	1	0	0	0	0	0	0	0
3%	2	2	2	1	1	1	1	0	0	0	0	0	0
4%	3	3	2	2	2	1	1	1	0	0	0	0	0
5%	4	3	3	2	2	2	1	1	0	0	0	0	0
6%	5	4	3	3	3	2	2	1	0	0	0	0	0
7%	5	5	4	4	3	2	2	1	1	0	0	0	0
8%	6	6	5	4	3	3	2	1	1	0	0	0	0
9%	7	6	5	5	4	3	2	2	1	0	0	0	0
10%	8	7	6	5	4	3	3	2	1	1	0	0	0
11%	9	8	7	6	5	4	3	2	1	1	0	0	0
12%	10	9	7	6	5	4	3	2	1	1	0	0	0
13%	10	9	8	7	6	4	3	2	1	1	0	0	0
14%	11	10	9	7	6	5	4	2	1	1	0	0	0
15%	12	11	9	8	6	5	4	3	1	1	0	0	0
16%	13	12	10	8	7	6	4	3	1	1	0	0	0
17%	14	13	11	9	7	6	4	3	1	1	0	0	0
18%	15	13	11	9	8	6	5	3	2	1	0	0	0
19%	16	14	12	10	8	7	5	3	2	1	0	0	0
20%	17	15	13	11	9	7	5	4	2	1	0	0	0
21%	18	16	13	11	9	7	6	4	2	1	0	0	0
22%	19	17	14	12	10	8	6	4	2	1	0	0	0
23%	20	18	15	12	10	8	6	4	2	1	0	0	0
24%	21	19	16	13	11	9	6	4	2	1	0	0	0
25%	22	20	16	14	11	9	7	5	2	1	0	0	0
26%	23	21	17	14	12	9	7	5	2	1	0	0	0
27%	25	22	18	15	12	10	7	5	2	1	0	0	0
28%	26	23	19	16	13	10	8	5	2	1	0	0	0
29%	27	24	20	16	13	11	8	5	3	2	0	0	0
30%	28	25	20	17	14	11	8	5	3	2	1	0	0
31%	29	26	21	18	14	11	9	6	3	2	1	0	0
32%	31	27	22	18	15	12	9	6	3	2	1	0	0
33%	32	28	23	19	16	12	9	6	3	2	1	0	0
34%	33	29	24	20	16	13	9	6	3	2	1	0	0
35%	34	30	25	20	17	13	10	6	3	2	1	0	0
36%	36	31	26	21	17	14	10	7	3	2	1	0	0
37%	37	32	26	22	18	14	10	7	3	2	1	0	0
38%	39	34	27	23	18	14	11	7	3	2	1	0	0
39%	40	35	28	23	19	15	11	7	3	2	1	0	0
40%	41	36	29	24	19	15	11	7	4	2	1	0	0
41%	43	37	30	25	20	16	12	8	4	2	1	0	0
42%	44	38	31	26	21	16	12	8	4	2	1	0	0
43%	46	40	32	26	21	17	12	8	4	2	1	0	0
44%	48	41	33	27	22	17	13	8	4	2	1	0	0
45%	49	42	34	28	22	18	13	8	4	2	1	0	0
46%	51	44	35	29	23	18	13	9	4	3	1	0	0
47%	53	45	36	29	24	19	14	9	4	3	1	0	0

**48%**	54	47	37	30	24	19	14	9	4	3	1	0	0
**49%**	56	48	38	31	25	20	14	9	4	3	1	0	0
**50%**	58	49	40	32	26	20	15	10	5	3	1	0	0
**51%**	60	51	41	33	26	21	15	10	5	3	1	0	0
**52%**	62	52	42	34	27	21	15	10	5	3	1	0	0
**53%**	64	54	43	35	28	22	16	10	5	3	1	0	0
**54%**	65	56	44	36	28	22	16	10	5	3	1	0	0
**55%**	68	57	45	36	29	23	17	11	5	3	1	0	0
**56%**	70	59	47	37	30	23	17	11	5	3	1	0	0
**57%**	72	61	48	38	30	24	17	11	5	3	1	0	0
**58%**	74	62	49	39	31	24	18	11	5	3	1	0	0
**59%**	76	64	50	40	32	25	18	12	5	3	1	0	0
**60%**	79	66	52	41	33	25	18	12	6	3	1	0	0
**61%**	81	68	53	42	33	26	19	12	6	3	1	0	0
**62%**	83	70	54	43	34	26	19	12	6	4	1	0	0
**63%**	86	72	56	44	35	27	20	12	6	4	1	0	0
**64%**	88	74	57	45	36	27	20	13	6	4	1	0	0
**65%**	91	76	58	46	36	28	20	13	6	4	1	0	0
**66%**	94	78	60	47	37	29	21	13	6	4	1	0	0
**67%**	97	80	61	48	38	29	21	13	6	4	1	0	0
**68%**	99	82	63	49	39	30	22	14	6	4	1	0	0
**69%**	102	84	64	51	40	30	22	14	7	4	1	0	0
**70%**	106	87	66	52	40	31	22	14	7	4	1	0	0
**71%**	109	89	68	53	41	31	23	14	7	4	1	0	0
**72%**	112	91	69	54	42	32	23	15	7	4	1	0	0
**73%**	115	94	71	55	43	33	24	15	7	4	1	0	0
**74%**	119	96	73	56	44	33	24	15	7	4	1	0	0
**75%**	122	99	74	58	45	34	24	15	7	4	1	0	0
**76%**	126	102	76	59	46	34	25	16	7	4	1	0	0
**77%**	130	104	78	60	46	35	25	16	8	4	1	0	0
**78%**	134	107	80	61	47	36	26	16	8	5	1	0	0
**79%**	138	110	82	63	48	36	26	17	8	5	1	0	0
**80%**	142	113	84	64	49	37	27	17	8	5	1	0	0
**81%**	147	116	86	65	50	38	27	17	8	5	1	0	0
**82%**	151	119	88	67	51	38	27	17	8	5	1	0	0
**83%**	156	123	90	68	52	39	28	18	8	5	2	0	0
**84%**	161	126	92	70	53	40	28	18	8	5	2	0	0
**85%**	166	130	94	71	54	40	29	18	9	5	2	0	0
**86%**	172	133	96	73	55	41	29	18	9	5	2	0	0
**87%**	177	137	98	74	56	42	30	19	9	5	2	0	0
**88%**	183	141	101	76	57	43	30	19	9	5	2	0	0
**89%**	189	145	103	77	58	43	31	19	9	5	2	0	0
**90%**	196	149	106	79	59	44	31	19	9	5	2	0	0
**91%**	202	153	108	80	60	45	32	20	9	5	2	0	0
**92%**	209	158	111	82	62	46	32	20	10	5	2	0	0
**93%**	217	162	113	84	63	46	33	20	10	6	2	0	0
**94%**	224	167	116	85	64	47	33	21	10	6	2	0	0
**95%**	232	172	119	87	65	48	34	21	10	6	2	0	0
**96%**	241	177	122	89	66	49	34	21	10	6	2	0	0
**97%**	250	182	125	91	67	49	35	22	10	6	2	0	0
**98%**	259	188	128	93	69	50	35	22	10	6	2	0	0
**99%**	269	194	131	95	70	51	36	22	10	6	2	0	0
**100%**	280	200	134	97	71	52	36	22	11	6	2	0	0

Supplementary Table S11. Differences between AMs' consumption levels calculated by means of novel pediatric-adjusted methodology vs conventional ATC/DDD methodology for fluoroquinolones (base case), %

Share / Age group	1-11 month	1 year	2 years	3 years	4 years	5 years	6 years	7 years	8 years	9 years	10 years	11 years	12 years
1%	-	-	-	-	-	0	0	0	0	0	0	0	0
2%	-	-	-	-	-	1	0	0	0	0	0	0	0
3%	-	-	-	-	-	1	1	0	0	0	0	0	0
4%	-	-	-	-	-	1	1	0	0	0	0	0	0
5%	-	-	-	-	-	1	1	1	0	0	0	0	0
6%	-	-	-	-	-	2	1	1	0	0	0	0	0
7%	-	-	-	-	-	2	1	1	0	0	0	0	0
8%	-	-	-	-	-	2	2	1	0	0	0	0	0
9%	-	-	-	-	-	3	2	1	0	0	0	0	0
10%	-	-	-	-	-	3	2	1	0	0	0	0	0
11%	-	-	-	-	-	3	2	1	0	0	0	0	0
12%	-	-	-	-	-	4	2	1	0	0	0	0	0
13%	-	-	-	-	-	4	3	1	0	0	0	0	0
14%	-	-	-	-	-	4	3	2	0	0	0	0	0
15%	-	-	-	-	-	4	3	2	0	0	0	0	0
16%	-	-	-	-	-	5	3	2	0	0	0	0	0
17%	-	-	-	-	-	5	4	2	0	0	0	0	0
18%	-	-	-	-	-	5	4	2	0	0	0	0	0
19%	-	-	-	-	-	6	4	2	0	0	0	0	0
20%	-	-	-	-	-	6	4	2	0	0	0	0	0
21%	-	-	-	-	-	6	4	2	0	0	0	0	0
22%	-	-	-	-	-	7	5	3	0	0	0	0	0
23%	-	-	-	-	-	7	5	3	0	0	0	0	0
24%	-	-	-	-	-	7	5	3	0	0	0	0	0
25%	-	-	-	-	-	8	5	3	0	0	0	0	0
26%	-	-	-	-	-	8	6	3	0	0	0	0	0
27%	-	-	-	-	-	8	6	3	0	0	0	0	0
28%	-	-	-	-	-	9	6	3	0	0	0	0	0
29%	-	-	-	-	-	9	6	3	0	0	0	0	0
30%	-	-	-	-	-	9	6	3	1	0	0	0	0
31%	-	-	-	-	-	10	7	4	1	0	0	0	0
32%	-	-	-	-	-	10	7	4	1	0	0	0	0
33%	-	-	-	-	-	10	7	4	1	0	0	0	0
34%	-	-	-	-	-	11	7	4	1	0	0	0	0
35%	-	-	-	-	-	11	8	4	1	0	0	0	0
36%	-	-	-	-	-	11	8	4	1	0	0	0	0
37%	-	-	-	-	-	12	8	4	1	0	0	0	0
38%	-	-	-	-	-	12	8	4	1	0	0	0	0
39%	-	-	-	-	-	12	9	5	1	0	0	0	0
40%	-	-	-	-	-	13	9	5	1	0	0	0	0
41%	-	-	-	-	-	13	9	5	1	0	0	0	0
42%	-	-	-	-	-	14	9	5	1	0	0	0	0
43%	-	-	-	-	-	14	9	5	1	0	0	0	0
44%	-	-	-	-	-	14	10	5	1	0	0	0	0
45%	-	-	-	-	-	15	10	5	1	0	0	0	0
46%	-	-	-	-	-	15	10	5	1	0	0	0	0
47%	-	-	-	-	-	15	10	6	1	0	0	0	0

**48%**	-	-	-	-	-	16	11	6	1	0	0	0	0
**49%**	-	-	-	-	-	16	11	6	1	0	0	0	0
**50%**	-	-	-	-	-	17	11	6	1	0	0	0	0
**51%**	-	-	-	-	-	17	11	6	1	0	0	0	0
**52%**	-	-	-	-	-	17	12	6	1	0	0	0	0
**53%**	-	-	-	-	-	18	12	6	1	0	0	0	0
**54%**	-	-	-	-	-	18	12	6	1	0	0	0	0
**55%**	-	-	-	-	-	19	12	7	1	0	0	0	0
**56%**	-	-	-	-	-	19	13	7	1	0	0	0	0
**57%**	-	-	-	-	-	19	13	7	1	0	0	0	0
**58%**	-	-	-	-	-	20	13	7	1	0	0	0	0
**59%**	-	-	-	-	-	20	14	7	1	0	0	0	0
**60%**	-	-	-	-	-	21	14	7	1	0	0	0	0
**61%**	-	-	-	-	-	21	14	7	1	0	0	0	0
**62%**	-	-	-	-	-	21	14	7	1	0	0	0	0
**63%**	-	-	-	-	-	22	15	8	1	0	0	0	0
**64%**	-	-	-	-	-	22	15	8	1	0	0	0	0
**65%**	-	-	-	-	-	23	15	8	1	0	0	0	0
**66%**	-	-	-	-	-	23	15	8	1	0	0	0	0
**67%**	-	-	-	-	-	24	16	8	1	0	0	0	0
**68%**	-	-	-	-	-	24	16	8	1	0	0	0	0
**69%**	-	-	-	-	-	24	16	8	1	0	0	0	0
**70%**	-	-	-	-	-	25	16	9	1	0	0	0	0
**71%**	-	-	-	-	-	25	17	9	1	0	0	0	0
**72%**	-	-	-	-	-	26	17	9	1	0	0	0	0
**73%**	-	-	-	-	-	26	17	9	1	0	0	0	0
**74%**	-	-	-	-	-	27	18	9	1	0	0	0	0
**75%**	-	-	-	-	-	27	18	9	1	0	0	0	0
**76%**	-	-	-	-	-	28	18	9	1	0	0	0	0
**77%**	-	-	-	-	-	28	18	9	1	0	0	0	0
**78%**	-	-	-	-	-	28	19	10	1	0	0	0	0
**79%**	-	-	-	-	-	29	19	10	1	0	0	0	0
**80%**	-	-	-	-	-	29	19	10	1	0	0	0	0
**81%**	-	-	-	-	-	30	20	10	1	0	0	0	0
**82%**	-	-	-	-	-	30	20	10	1	0	0	0	0
**83%**	-	-	-	-	-	31	20	10	1	0	0	0	0
**84%**	-	-	-	-	-	31	20	10	1	0	0	0	0
**85%**	-	-	-	-	-	32	21	11	1	0	0	0	0
**86%**	-	-	-	-	-	32	21	11	1	0	0	0	0
**87%**	-	-	-	-	-	33	21	11	2	0	0	0	0
**88%**	-	-	-	-	-	33	22	11	2	0	0	0	0
**89%**	-	-	-	-	-	34	22	11	2	0	0	0	0
**90%**	-	-	-	-	-	34	22	11	2	0	0	0	0
**91%**	-	-	-	-	-	35	22	11	2	0	0	0	0
**92%**	-	-	-	-	-	35	23	11	2	0	0	0	0
**93%**	-	-	-	-	-	36	23	12	2	0	0	0	0
**94%**	-	-	-	-	-	37	23	12	2	0	0	0	0
**95%**	-	-	-	-	-	37	24	12	2	0	0	0	0
**96%**	-	-	-	-	-	38	24	12	2	0	0	0	0
**97%**	-	-	-	-	-	38	24	12	2	0	0	0	0
**98%**	-	-	-	-	-	39	25	12	2	0	0	0	0
**99%**	-	-	-	-	-	39	25	12	2	0	0	0	0
**100%**	-	-	-	-	-	40	25	13	2	0	0	0	0

Supplementary Table S12. Differences between AMs' consumption levels calculated by means of novel pediatric-adjusted methodology vs conventional ATC/DDD methodology for macrolides (base case), %

Share / Age group	1-11 month	1 year	2 years	3 years	4 years	5 years	6 years	7 years	8 years	9 years	10 years	11 years	12 years
1%	1	1	1	0	0	0	0	0	0	0	0	0	0
2%	1	1	1	1	1	1	1	0	0	0	0	0	0
3%	2	2	2	1	1	1	1	1	0	0	0	0	0
4%	3	2	2	2	2	1	1	1	1	0	0	0	0
5%	3	3	3	2	2	2	1	1	1	0	0	0	0
6%	4	4	3	3	2	2	2	1	1	0	0	0	0
7%	5	4	4	3	3	2	2	2	1	0	0	0	0
8%	6	5	4	4	3	3	2	2	1	0	0	0	0
9%	6	6	5	4	4	3	3	2	1	0	0	0	0
10%	7	6	6	5	4	4	3	2	1	1	0	0	0
11%	8	7	6	5	5	4	3	2	2	1	0	0	0
12%	9	8	7	6	5	4	3	3	2	1	0	0	0
13%	9	9	7	6	5	5	4	3	2	1	0	0	0
14%	10	9	8	7	6	5	4	3	2	1	0	0	0
15%	11	10	9	7	6	5	4	3	2	1	0	0	0
16%	12	11	9	8	7	6	5	3	2	1	0	0	0
17%	13	12	10	8	7	6	5	4	2	1	0	0	0
18%	14	12	11	9	8	7	5	4	3	1	0	0	0
19%	14	13	11	10	8	7	6	4	3	1	0	0	0
20%	15	14	12	10	9	7	6	4	3	1	0	0	0
21%	16	15	13	11	9	8	6	5	3	1	0	0	0
22%	17	15	13	11	10	8	6	5	3	1	0	0	0
23%	18	16	14	12	10	8	7	5	3	1	0	0	0
24%	19	17	15	12	11	9	7	5	3	1	0	0	0
25%	20	18	15	13	11	9	7	6	4	1	0	0	0
26%	21	19	16	14	12	10	8	6	4	1	0	0	0
27%	22	20	17	14	12	10	8	6	4	1	0	0	0
28%	23	21	18	15	13	11	8	6	4	2	0	0	0
29%	24	22	18	16	13	11	9	7	4	2	0	0	0
30%	25	23	19	16	14	11	9	7	4	2	0	0	0
31%	26	24	20	17	14	12	9	7	4	2	0	0	0
32%	27	25	21	18	15	12	10	7	5	2	0	0	0
33%	29	26	21	18	15	13	10	7	5	2	0	0	0
34%	30	27	22	19	16	13	10	8	5	2	0	0	0
35%	31	28	23	20	16	14	11	8	5	2	0	0	0
36%	32	29	24	20	17	14	11	8	5	2	0	0	0
37%	33	30	25	21	18	15	11	8	5	2	0	0	0
38%	35	31	26	22	18	15	12	9	5	2	0	0	0
39%	36	32	27	22	19	15	12	9	6	2	0	0	0
40%	37	33	27	23	19	16	12	9	6	2	0	0	0
41%	39	34	28	24	20	16	13	10	6	2	0	0	0
42%	40	35	29	25	21	17	13	10	6	2	0	0	0
43%	42	37	30	25	21	17	14	10	6	2	0	0	0
44%	43	38	31	26	22	18	14	10	6	2	0	0	0
45%	44	39	32	27	22	18	14	11	7	2	0	0	0
46%	46	40	33	28	23	19	15	11	7	3	0	0	0
47%	48	42	34	28	24	19	15	11	7	3	0	0	0

**48%**	49	43	35	29	24	20	15	11	7	3	0	0	0
**49%**	51	44	36	30	25	20	16	12	7	3	0	0	0
**50%**	52	46	37	31	26	21	16	12	7	3	0	0	0
**51%**	54	47	38	32	26	21	17	12	7	3	0	0	0
**52%**	56	48	40	33	27	22	17	12	8	3	0	0	0
**53%**	58	50	41	33	28	22	17	13	8	3	0	0	0
**54%**	59	51	42	34	28	23	18	13	8	3	0	0	0
**55%**	61	53	43	35	29	24	18	13	8	3	0	0	0
**56%**	63	55	44	36	30	24	19	14	8	3	0	0	0
**57%**	65	56	45	37	30	25	19	14	8	3	0	0	0
**58%**	67	58	47	38	31	25	19	14	9	3	0	0	0
**59%**	69	60	48	39	32	26	20	14	9	3	0	0	0
**60%**	72	61	49	40	33	26	20	15	9	3	0	0	0
**61%**	74	63	50	41	33	27	21	15	9	3	0	0	0
**62%**	76	65	52	42	34	28	21	15	9	3	0	0	0
**63%**	78	67	53	43	35	28	21	16	9	4	0	0	0
**64%**	81	69	54	44	36	29	22	16	10	4	0	0	0
**65%**	83	71	56	45	36	29	22	16	10	4	0	0	0
**66%**	86	72	57	46	37	30	23	16	10	4	0	0	0
**67%**	88	75	59	47	38	31	23	17	10	4	0	0	0
**68%**	91	77	60	48	39	31	24	17	10	4	0	0	0
**69%**	94	79	62	49	40	32	24	17	10	4	0	0	0
**70%**	97	81	63	51	41	32	25	18	11	4	0	0	0
**71%**	100	83	65	52	41	33	25	18	11	4	0	0	0
**72%**	103	86	67	53	42	34	25	18	11	4	0	0	0
**73%**	106	88	68	54	43	34	26	19	11	4	0	0	0
**74%**	109	91	70	55	44	35	26	19	11	4	0	0	0
**75%**	113	93	72	56	45	36	27	19	11	4	0	0	0
**76%**	116	96	73	58	46	36	27	20	12	4	0	0	0
**77%**	120	98	75	59	47	37	28	20	12	4	0	0	0
**78%**	124	101	77	60	48	38	28	20	12	4	0	0	0
**79%**	128	104	79	62	49	38	29	20	12	4	0	0	0
**80%**	132	107	81	63	50	39	29	21	12	4	0	0	0
**81%**	136	110	83	64	51	40	30	21	13	5	0	0	0
**82%**	140	113	85	66	52	41	30	21	13	5	0	0	0
**83%**	145	116	87	67	53	41	31	22	13	5	0	0	0
**84%**	150	120	89	69	54	42	31	22	13	5	0	0	0
**85%**	155	123	91	70	55	43	32	22	13	5	0	0	0
**86%**	160	127	93	72	56	44	32	23	13	5	0	0	0
**87%**	166	130	96	73	57	44	33	23	14	5	0	0	0
**88%**	171	134	98	75	58	45	33	23	14	5	0	0	0
**89%**	177	138	101	76	59	46	34	24	14	5	0	0	0
**90%**	183	142	103	78	60	47	34	24	14	5	0	0	0
**91%**	190	147	106	80	61	48	35	24	14	5	0	0	0
**92%**	197	151	108	81	63	48	35	25	15	5	0	0	0
**93%**	204	156	111	83	64	49	36	25	15	5	0	0	0
**94%**	211	160	114	85	65	50	37	25	15	5	0	0	0
**95%**	219	165	116	87	66	51	37	26	15	5	0	0	0
**96%**	228	171	119	89	68	52	38	26	15	5	0	0	0
**97%**	237	176	122	90	69	53	38	27	15	6	0	0	0
**98%**	246	182	126	92	70	53	39	27	16	6	0	0	0
**99%**	256	188	129	94	71	54	39	27	16	6	0	0	0
**100%**	267	194	132	96	73	55	40	28	16	6	0	0	0

Supplementary Table S13. Differences between AMs' consumption levels calculated by means of novel pediatric-adjusted methodology vs conventional ATC/DDD methodology for lincosamides (base case), %

Share / Age group	1-11 month	1 year	2 years	3 years	4 years	5 years	6 years	7 years	8 years	9 years	10 years	11 years	12 years
**1%**	1	1	1	1	1	1	1	1	1	1	1	1	1
**2%**	2	2	2	2	2	2	2	2	2	1	1	1	1
**3%**	3	3	3	3	3	2	2	2	2	2	2	2	2
**4%**	4	4	4	4	3	3	3	3	3	3	3	2	2
**5%**	5	5	5	4	4	4	4	4	4	4	3	3	3
**6%**	6	6	6	5	5	5	5	5	5	4	4	4	3
**7%**	7	7	6	6	6	6	6	6	5	5	5	4	4
**8%**	8	8	8	7	7	7	7	6	6	6	6	5	5
**9%**	9	9	8	8	8	8	8	7	7	7	6	6	5
**10%**	10	10	10	9	9	9	8	8	8	7	7	6	6
**11%**	11	11	11	10	10	10	9	9	9	8	8	7	6
**12%**	12	12	12	11	11	11	10	10	10	9	8	8	7
**13%**	14	13	13	12	12	12	11	11	10	10	9	9	8
**14%**	15	14	14	13	13	13	12	12	11	11	10	9	8
**15%**	16	16	15	14	14	14	13	13	12	12	11	10	9
**16%**	17	17	16	16	15	15	14	14	13	12	12	11	10
**17%**	19	18	17	17	16	16	15	15	14	13	12	12	10
**18%**	20	19	19	18	17	17	16	16	15	14	13	12	11
**19%**	21	21	20	19	18	18	17	17	16	15	14	13	12
**20%**	23	22	21	20	20	19	18	18	17	16	15	14	12
**21%**	24	23	22	22	21	20	19	19	18	17	16	15	13
**22%**	25	25	24	23	22	21	21	20	19	18	17	15	14
**23%**	27	26	25	24	23	22	22	21	20	19	18	16	15
**24%**	28	27	26	25	24	24	23	22	21	20	18	17	15
**25%**	30	29	28	27	26	25	24	23	22	21	19	18	16
**26%**	31	30	29	28	27	26	25	24	23	22	20	19	17
**27%**	33	32	31	29	28	27	26	25	24	23	21	20	18
**28%**	35	34	32	31	30	29	28	26	25	24	22	20	18
**29%**	36	35	34	32	31	30	29	28	26	25	23	21	19
**30%**	38	37	35	34	33	31	30	29	27	26	24	22	20
**31%**	40	39	37	35	34	33	31	30	29	27	25	23	21
**32%**	42	40	38	37	36	34	33	31	30	28	26	24	22
**33%**	44	42	40	39	37	36	34	33	31	29	27	25	22
**34%**	45	44	42	40	39	37	36	34	32	30	28	26	23
**35%**	47	46	44	42	40	39	37	35	34	32	29	27	24
**36%**	49	48	46	44	42	40	39	37	35	33	31	28	25
**37%**	52	50	47	45	43	42	40	38	36	34	32	29	26
**38%**	54	52	49	47	45	43	42	40	38	35	33	30	27
**39%**	56	54	51	49	47	45	43	41	39	37	34	31	28
**40%**	58	56	53	51	49	47	45	43	40	38	35	32	29
**41%**	60	58	55	53	51	49	46	44	42	39	36	33	29
**42%**	63	60	57	55	53	50	48	46	43	41	37	34	30
**43%**	65	63	60	57	54	52	50	47	45	42	39	35	31
**44%**	68	65	62	59	56	54	52	49	46	43	40	36	32
**45%**	71	68	64	61	58	56	53	51	48	45	41	37	33
**46%**	73	70	67	63	61	58	55	52	49	46	43	39	34
**47%**	76	73	69	66	63	60	57	54	51	48	44	40	35

**48%**	79	76	72	68	65	62	59	56	53	49	45	41	36
**49%**	82	78	74	70	67	64	61	58	55	51	47	42	37
**50%**	85	81	77	73	69	66	63	60	56	52	48	43	38
**51%**	88	84	80	76	72	69	65	62	58	54	49	45	39
**52%**	92	88	82	78	74	71	67	64	60	56	51	46	41
**53%**	95	91	85	81	77	73	69	66	62	57	52	47	42
**54%**	98	94	88	84	80	76	72	68	64	59	54	49	43
**55%**	102	97	91	87	82	78	74	70	66	61	56	50	44
**56%**	106	101	95	90	85	81	76	72	67	63	57	51	45
**57%**	110	105	98	93	88	83	79	74	70	64	59	53	46
**58%**	114	109	102	96	91	86	81	77	72	66	60	54	47
**59%**	118	112	105	99	94	89	84	79	74	68	62	56	49
**60%**	123	117	109	102	97	92	87	81	76	70	64	57	50
**61%**	128	121	113	106	100	95	89	84	78	72	66	59	51
**62%**	132	125	117	110	103	98	92	86	81	74	67	60	52
**63%**	138	130	121	113	107	101	95	89	83	76	69	62	54
**64%**	143	135	125	117	110	104	98	92	85	78	71	63	55
**65%**	148	140	130	121	114	108	101	95	88	81	73	65	56
**66%**	154	145	134	125	118	111	104	98	90	83	75	67	58
**67%**	160	151	139	130	122	115	108	100	93	85	77	68	59
**68%**	167	156	144	134	126	118	111	104	96	88	79	70	61
**69%**	173	163	150	139	130	122	115	107	99	90	81	72	62
**70%**	180	169	155	144	135	126	118	110	102	93	83	74	63
**71%**	188	176	161	149	139	130	122	113	104	95	86	76	65
**72%**	196	183	167	155	144	135	126	117	108	98	88	77	67
**73%**	204	190	173	160	149	139	130	120	111	101	90	79	68
**74%**	212	198	180	166	154	144	134	124	114	103	92	81	70
**75%**	222	206	187	172	160	149	138	128	117	106	95	83	71
**76%**	232	214	194	178	165	154	143	132	121	109	97	85	73
**77%**	242	224	202	185	171	159	147	136	124	112	100	88	75
**78%**	253	233	210	192	177	164	152	140	128	115	103	90	76
**79%**	265	243	218	200	184	170	157	145	132	119	105	92	78
**80%**	278	254	228	207	191	176	163	149	136	122	108	94	80
**81%**	291	266	237	216	198	182	168	154	140	125	111	97	82
**82%**	306	278	247	224	205	189	174	159	144	129	114	99	83
**83%**	321	292	258	233	213	196	180	164	149	133	117	101	85
**84%**	338	306	270	243	221	203	186	169	153	136	120	104	87
**85%**	357	321	282	253	230	210	192	175	158	140	123	106	89
**86%**	377	338	295	264	239	218	199	180	163	144	126	109	91
**87%**	399	356	309	275	249	226	206	186	168	149	130	112	93
**88%**	423	375	324	288	259	235	213	193	173	153	133	114	95
**89%**	449	396	340	301	270	244	221	199	178	157	137	117	98
**90%**	478	420	358	315	282	254	229	206	184	162	141	120	100
**91%**	511	445	377	330	294	264	238	213	190	167	144	123	102
**92%**	547	473	397	346	307	275	247	221	196	172	148	126	104
**93%**	588	504	420	363	321	287	257	229	203	177	152	129	107
**94%**	634	539	444	382	336	299	267	237	209	182	157	133	109
**95%**	687	577	471	403	352	312	277	246	216	188	161	136	111
**96%**	749	621	501	425	370	326	289	255	224	194	165	139	114
**97%**	820	671	534	449	389	341	301	265	231	200	170	143	117
**98%**	905	728	571	476	409	357	314	275	240	206	175	147	119
**99%**	1008	795	613	505	431	374	327	286	248	213	180	150	122
**100%**	1133	873	660	538	455	393	342	297	257	220	185	154	125

Supplementary Table S14. Differences between AMs' consumption levels calculated by means of novel pediatric-adjusted methodology vs conventional ATC/DDD methodology for glycopeptides (base case), %

Share / Age group	1-11 month	1 year	2 years	3 years	4 years	5 years	6 years	7 years	8 years	9 years	10 years	11 years	12 years
1%	1	1	1	1	1	1	1	1	0	0	0	0	0
2%	2	2	2	1	1	1	1	1	1	1	1	1	0
3%	3	2	2	2	2	2	2	2	2	1	1	1	1
4%	3	3	3	3	3	3	2	2	2	2	2	1	1
5%	4	4	4	4	3	3	3	3	2	2	2	1	1
6%	5	5	5	4	4	4	4	3	3	3	2	2	1
7%	6	6	6	5	5	5	4	4	4	3	3	2	1
8%	7	7	7	6	6	5	5	5	4	4	3	2	2
9%	8	8	7	7	7	6	6	5	5	4	3	3	2
10%	9	9	8	8	7	7	6	6	5	5	4	3	2
11%	10	10	9	9	8	7	7	6	6	5	4	3	2
12%	11	11	10	9	9	8	8	7	6	6	5	4	2
13%	12	12	11	10	10	9	8	8	7	6	5	4	3
14%	14	13	12	11	10	10	9	8	7	7	5	4	3
15%	15	14	13	12	11	11	10	9	8	7	6	5	3
16%	16	15	14	13	12	11	11	10	9	7	6	5	3
17%	17	16	15	14	13	12	11	10	9	8	7	5	3
18%	18	17	16	15	14	13	12	11	10	8	7	6	4
19%	19	18	17	16	15	14	13	12	10	9	7	6	4
20%	21	20	18	17	16	15	13	12	11	10	8	6	4
21%	22	21	19	18	16	15	14	13	12	10	8	7	4
22%	23	22	20	19	17	16	15	14	12	11	9	7	5
23%	24	23	21	20	18	17	16	14	13	11	9	7	5
24%	26	24	22	21	19	18	17	15	13	12	10	7	5
25%	27	26	24	22	20	19	17	16	14	12	10	8	5
26%	28	27	25	23	21	20	18	17	15	13	11	8	5
27%	30	28	26	24	22	21	19	17	15	13	11	9	6
28%	31	29	27	25	23	22	20	18	16	14	11	9	6
29%	33	31	28	26	24	23	21	19	17	15	12	9	6
30%	34	32	30	27	25	24	22	20	17	15	12	10	6
31%	36	34	31	29	26	24	23	20	18	16	13	10	7
32%	38	35	32	30	28	25	23	21	19	16	13	10	7
33%	39	37	34	31	29	26	24	22	20	17	14	11	7
34%	41	38	35	32	30	28	25	23	20	17	14	11	7
35%	43	40	36	33	31	28	26	24	21	18	15	11	7
36%	44	41	38	35	32	30	27	24	22	19	15	12	8
37%	46	43	39	36	33	31	28	25	22	19	16	12	8
38%	48	45	41	37	35	32	29	26	23	20	16	12	8
39%	50	47	42	39	36	33	30	27	24	20	17	13	8
40%	52	48	44	40	37	34	31	28	25	21	17	13	9
41%	54	50	46	42	38	35	32	29	25	22	18	14	9
42%	56	52	47	43	40	36	33	30	26	22	18	14	9
43%	58	54	49	45	41	37	34	31	27	23	19	14	9
44%	60	56	50	46	42	39	35	32	28	24	19	15	10
45%	62	58	52	48	44	40	36	33	29	24	20	15	10
46%	65	60	54	49	45	41	37	34	29	25	20	15	10
47%	67	62	56	51	46	42	39	35	30	26	21	16	10

**48%**	69	64	58	53	48	44	40	36	31	27	21	16	11
**49%**	72	66	60	54	50	45	41	37	32	27	22	17	11
**50%**	74	69	62	56	51	46	42	38	33	28	23	17	11
**51%**	77	71	64	58	53	48	43	39	34	29	23	17	11
**52%**	80	73	66	59	54	49	45	40	35	29	24	18	11
**53%**	83	76	68	61	56	51	46	41	36	30	24	18	12
**54%**	85	79	70	63	57	52	47	42	37	31	25	19	12
**55%**	89	81	72	65	59	54	48	43	37	32	25	19	12
**56%**	92	84	75	67	61	55	50	44	38	32	26	20	12
**57%**	95	87	77	69	63	57	51	45	39	33	27	20	13
**58%**	98	90	79	71	64	58	52	46	40	34	27	20	13
**59%**	102	93	82	73	66	60	54	48	41	35	28	21	13
**60%**	105	96	84	76	68	62	55	49	42	36	28	21	14
**61%**	109	99	87	78	70	63	57	50	43	36	29	22	14
**62%**	112	102	90	80	72	65	58	51	44	37	30	22	14
**63%**	116	106	93	83	74	67	60	53	45	38	30	23	14
**64%**	120	109	95	85	76	68	61	54	46	39	31	23	15
**65%**	125	113	98	87	78	70	63	55	48	40	32	23	15
**66%**	129	116	101	90	80	72	64	56	49	41	32	24	15
**67%**	134	120	105	93	83	74	66	58	50	41	33	24	15
**68%**	138	124	108	95	85	76	67	59	51	42	33	25	16
**69%**	143	128	111	98	87	78	69	61	52	43	34	25	16
**70%**	149	133	115	101	90	80	71	62	53	44	35	26	16
**71%**	154	137	118	104	92	82	73	63	54	45	35	26	16
**72%**	160	142	122	107	95	84	74	65	55	46	36	27	17
**73%**	165	147	126	110	97	86	76	66	57	47	37	27	17
**74%**	172	152	130	113	100	89	78	68	58	48	37	28	17
**75%**	178	157	134	117	103	91	80	70	59	49	38	28	18
**76%**	185	163	138	120	106	93	82	71	60	50	39	28	18
**77%**	192	168	142	124	108	96	84	73	62	51	40	29	18
**78%**	199	174	147	127	111	98	86	74	63	52	40	29	18
**79%**	207	181	152	131	115	101	88	76	64	53	41	30	19
**80%**	216	187	157	135	118	103	90	78	66	54	42	30	19
**81%**	224	194	162	139	121	106	92	80	67	55	43	31	19
**82%**	234	201	167	143	124	109	95	81	68	56	43	31	20
**83%**	243	209	173	147	128	111	97	83	70	57	44	32	20
**84%**	254	217	178	152	131	114	99	85	71	58	45	33	20
**85%**	265	225	185	157	135	117	102	87	73	59	46	33	20
**86%**	276	234	191	161	139	120	104	89	74	60	46	33	21
**87%**	289	244	197	166	143	123	107	91	76	61	47	34	21
**88%**	302	254	204	172	147	127	109	93	77	62	48	35	21
**89%**	317	264	211	177	151	130	112	95	79	64	49	35	21
**90%**	332	275	219	183	155	133	114	97	81	65	50	36	22
**91%**	349	287	227	188	160	137	117	99	82	66	50	36	22
**92%**	366	300	235	194	164	140	120	101	84	67	51	37	22
**93%**	386	313	244	201	169	144	123	103	86	68	52	37	23
**94%**	407	327	254	207	174	148	126	106	87	70	53	38	23
**95%**	430	343	263	215	179	152	129	108	89	71	54	38	23
**96%**	455	359	274	222	185	156	132	111	91	72	55	39	24
**97%**	483	377	285	229	190	160	135	113	93	74	56	39	24
**98%**	513	397	296	237	196	165	139	115	94	75	57	40	24
**99%**	547	418	309	246	202	169	142	118	96	76	58	41	24
**100%**	585	440	322	254	209	174	146	121	98	78	58	41	25

Supplementary Table S15. Differences between AMs' consumption levels calculated by means of novel pediatric-adjusted methodology vs conventional ATC/DDD methodology for co-trimoxazole (base case), %

Share / Age group	1-11 month	1 year	2 years	3 years	4 years	5 years	6 years	7 years	8 years	9 years	10 years	11 years	12 years
1%	1	1	1	1	1	1	1	1	1	0	0	0	0
2%	2	2	1	1	1	1	1	1	1	1	1	1	1
3%	3	3	2	2	2	2	2	2	2	1	1	1	1
4%	4	3	3	3	3	3	3	2	2	2	2	1	1
5%	5	4	4	4	4	3	3	3	3	2	2	2	1
6%	5	5	5	5	4	4	4	4	3	3	3	2	1
7%	6	6	6	5	5	5	5	4	4	3	3	2	2
8%	7	7	6	6	6	6	5	5	4	4	3	3	2
9%	8	8	7	7	7	6	6	5	5	4	4	3	2
10%	9	9	8	8	7	7	6	6	6	5	4	3	3
11%	10	10	9	9	8	8	7	7	6	5	5	4	3
12%	12	11	10	10	9	8	8	7	7	6	5	4	3
13%	13	12	11	10	10	9	9	8	7	6	6	5	3
14%	14	13	12	11	11	10	9	9	8	7	6	5	4
15%	15	14	13	12	12	11	10	9	8	8	6	5	4
16%	16	15	14	13	12	12	11	10	9	8	7	6	4
17%	17	16	15	14	13	12	12	11	10	9	7	6	4
18%	18	18	16	15	14	13	12	12	10	9	8	6	5
19%	19	19	17	16	15	14	13	12	11	10	8	7	5
20%	21	20	18	17	16	15	14	13	12	10	9	7	5
21%	22	21	19	18	17	16	15	14	12	11	9	8	5
22%	23	22	21	19	18	17	16	14	13	12	10	8	6
23%	25	23	22	20	19	18	17	15	14	12	10	8	6
24%	26	25	23	21	20	19	17	16	14	13	11	9	6
25%	27	26	24	22	21	20	18	17	15	13	11	9	7
26%	29	27	25	24	22	21	19	18	16	14	12	10	7
27%	30	29	27	25	23	21	20	18	17	15	12	10	7
28%	32	30	28	26	24	23	21	19	17	15	13	10	7
29%	33	31	29	27	25	23	22	20	18	16	13	11	8
30%	35	33	30	28	26	25	23	21	19	16	14	11	8
31%	36	34	32	29	27	26	24	22	19	17	14	12	8
32%	38	36	33	31	29	27	25	23	20	18	15	12	9
33%	40	37	34	32	30	28	26	23	21	18	16	12	9
34%	42	39	36	33	31	29	27	24	22	19	16	13	9
35%	43	41	37	35	32	30	28	25	23	20	17	13	10
36%	45	42	39	36	33	31	29	26	23	20	17	14	10
37%	47	44	40	37	35	32	30	27	24	21	18	14	10
38%	49	46	42	39	36	33	31	28	25	22	18	15	10
39%	51	47	44	40	37	34	32	29	26	23	19	15	11
40%	53	49	45	42	39	36	33	30	27	23	19	16	11
41%	55	51	47	43	40	37	34	31	28	24	20	16	11
42%	57	53	48	45	41	38	35	32	28	25	21	16	12
43%	59	55	50	46	43	39	36	33	29	25	21	17	12
44%	61	57	52	48	44	41	37	34	30	26	22	17	12
45%	63	59	54	49	45	42	38	35	31	27	22	18	13
46%	66	61	56	51	47	43	40	36	32	28	23	18	13
47%	68	64	58	53	49	45	41	37	33	29	24	19	13

**48%**	71	66	60	55	50	46	42	38	34	29	24	19	14
**49%**	73	68	62	56	52	47	43	39	35	30	25	20	14
**50%**	76	70	64	58	53	49	45	40	36	31	26	20	14
**51%**	79	73	66	60	55	50	46	42	37	32	26	21	14
**52%**	81	75	68	62	57	52	47	43	38	32	27	21	15
**53%**	84	78	70	64	58	53	49	44	39	33	28	22	15
**54%**	87	81	72	66	60	55	50	45	40	34	28	22	16
**55%**	90	83	75	68	62	57	51	46	41	35	29	23	16
**56%**	94	86	77	70	64	58	53	47	42	36	30	23	16
**57%**	97	89	80	72	66	60	54	49	43	37	30	24	16
**58%**	100	92	82	74	68	62	56	50	44	38	31	24	17
**59%**	104	95	85	77	70	63	57	51	45	39	32	25	17
**60%**	107	98	87	79	72	65	59	53	46	40	32	25	18
**61%**	111	102	90	81	74	67	60	54	47	40	33	26	18
**62%**	115	105	93	84	76	69	62	55	49	41	34	26	18
**63%**	119	108	96	86	78	71	64	57	50	42	35	27	19
**64%**	123	112	99	89	80	73	65	58	51	43	35	27	19
**65%**	128	116	102	92	83	75	67	60	52	44	36	28	19
**66%**	132	120	105	94	85	77	69	61	53	45	37	29	20
**67%**	137	124	109	97	87	79	71	63	55	46	38	29	20
**68%**	142	128	112	100	90	81	72	64	56	47	38	30	20
**69%**	147	133	116	103	92	83	74	66	57	48	39	30	21
**70%**	153	137	119	106	95	85	76	67	58	49	40	31	21
**71%**	158	142	123	109	98	88	78	69	60	50	41	31	21
**72%**	164	147	127	112	101	90	80	71	61	51	42	32	22
**73%**	170	152	131	116	103	92	82	72	63	53	42	32	22
**74%**	177	157	135	119	106	95	84	74	64	54	43	33	23
**75%**	183	163	140	123	109	97	86	76	65	55	44	34	23
**76%**	190	169	144	127	112	100	89	78	67	56	45	34	23
**77%**	198	175	149	131	116	103	91	79	68	57	46	35	24
**78%**	206	181	154	134	119	105	93	81	70	58	47	36	24
**79%**	214	188	159	139	122	108	95	83	71	59	47	36	24
**80%**	223	195	164	143	126	111	98	85	73	61	48	37	25
**81%**	232	203	170	147	129	114	100	87	75	62	49	37	25
**82%**	242	210	176	152	133	117	103	89	76	63	50	38	26
**83%**	252	218	182	156	137	120	105	91	78	64	51	39	26
**84%**	263	227	188	162	141	123	108	94	79	66	52	39	26
**85%**	275	236	195	167	145	127	111	96	81	67	53	40	27
**86%**	288	246	202	172	149	130	113	98	83	68	54	41	27
**87%**	301	256	209	177	154	134	116	100	85	70	55	41	28
**88%**	316	267	217	183	158	137	119	103	87	71	56	42	28
**89%**	331	278	225	189	163	141	122	105	88	72	57	43	28
**90%**	348	290	233	196	168	145	125	107	90	74	58	43	29
**91%**	366	303	242	202	173	149	129	110	92	75	59	44	29
**92%**	385	317	251	209	178	153	132	112	94	77	60	45	30
**93%**	406	332	261	216	184	157	135	115	96	78	61	45	30
**94%**	430	348	271	224	189	162	139	118	98	80	62	46	31
**95%**	455	365	282	232	195	166	142	121	100	81	63	47	31
**96%**	483	384	294	240	201	171	146	123	103	83	64	48	31
**97%**	514	404	307	249	208	176	150	126	105	84	66	48	32
**98%**	548	426	320	258	215	181	154	129	107	86	67	49	32
**99%**	586	450	334	268	222	187	158	132	109	88	68	50	33
**100%**	630	476	349	278	229	192	162	136	112	90	69	51	33

Supplementary Table S16. Differences between AMs' consumption levels calculated by means of novel pediatric-adjusted methodology vs conventional ATC/DDD methodology for nitroimidazoles (base case), %

Share / Age group	1-11 month	1 year	2 years	3 years	4 years	5 years	6 years	7 years	8 years	9 years	10 years	11 years	12 years
1%	1	1	1	1	1	1	1	1	1	1	1	1	1
2%	2	2	2	2	2	1	1	1	1	1	1	1	1
3%	3	3	3	2	2	2	2	2	2	2	2	2	2
4%	4	4	3	3	3	3	3	3	3	3	3	3	2
5%	5	5	4	4	4	4	4	4	4	3	3	3	3
6%	6	5	5	5	5	5	5	4	4	4	4	4	4
7%	7	6	6	6	6	5	5	5	5	5	5	5	4
8%	8	7	7	7	6	6	6	6	6	6	5	5	5
9%	9	8	8	8	7	7	7	7	7	6	6	6	6
10%	10	9	9	9	8	8	8	8	7	7	7	7	6
11%	11	11	10	9	9	9	9	8	8	8	8	7	7
12%	12	12	11	11	10	10	10	9	9	9	8	8	8
13%	13	13	12	11	11	10	10	10	10	10	9	9	8
14%	14	14	13	12	12	11	11	11	11	10	10	10	9
15%	15	15	14	13	13	12	12	12	12	11	11	10	10
16%	17	16	15	14	14	13	13	13	12	12	12	11	10
17%	18	17	16	16	15	14	14	14	13	13	12	12	11
18%	19	18	17	17	16	15	15	15	14	14	13	13	12
19%	20	20	19	18	17	16	16	16	15	15	14	13	13
20%	22	21	20	19	18	17	17	17	16	16	15	14	13
21%	23	22	21	20	19	18	18	17	17	16	16	15	14
22%	24	23	22	21	20	19	19	18	18	17	17	16	15
23%	26	25	23	22	21	20	20	19	19	18	18	17	16
24%	27	26	25	23	22	21	21	21	20	19	18	18	17
25%	29	27	26	25	23	22	22	22	21	20	19	19	17
26%	30	29	27	26	25	23	23	23	22	21	20	19	18
27%	32	30	29	27	26	24	24	24	23	22	21	20	19
28%	33	32	30	28	27	25	25	25	24	23	22	21	20
29%	35	33	31	30	28	27	27	26	25	24	23	22	21
30%	36	35	33	31	29	28	28	27	26	25	24	23	22
31%	38	36	34	32	31	29	29	28	27	26	25	24	23
32%	40	38	36	34	32	30	30	29	28	27	26	25	24
33%	42	40	37	35	33	32	31	31	30	29	27	26	25
34%	43	41	39	37	35	33	33	32	31	30	28	27	26
35%	45	43	40	38	36	34	34	33	32	31	30	28	27
36%	47	45	42	40	37	35	35	34	33	32	31	29	28
37%	49	47	44	41	39	37	37	36	34	33	32	30	29
38%	51	49	45	43	40	38	38	37	36	34	33	31	30
39%	53	51	47	44	42	40	39	38	37	36	34	33	31
40%	55	53	49	46	43	41	41	40	38	37	35	34	32
41%	58	55	51	48	45	42	42	41	40	38	37	35	33
42%	60	57	53	50	47	44	44	42	41	39	38	36	34
43%	62	59	55	51	48	45	45	44	42	41	39	37	35
44%	64	61	57	53	50	47	47	45	44	42	40	38	36
45%	67	63	59	55	52	49	48	47	45	44	42	40	37
46%	69	66	61	57	53	50	50	48	47	45	43	41	39
47%	72	68	63	59	55	52	52	50	48	46	44	42	40

**48%**	75	71	65	61	57	53	53	52	50	48	46	44	41
**49%**	77	73	67	63	59	55	55	53	51	49	47	45	42
**50%**	80	76	70	65	61	57	57	55	53	51	49	46	44
**51%**	83	78	72	67	63	59	59	57	55	53	50	48	45
**52%**	86	81	75	70	65	61	61	59	56	54	52	49	46
**53%**	89	84	77	72	67	63	62	60	58	56	53	51	48
**54%**	93	87	80	74	69	64	64	62	60	58	55	52	49
**55%**	96	90	83	77	71	67	66	64	62	59	57	54	51
**56%**	100	93	85	79	74	69	68	66	64	61	58	55	52
**57%**	103	96	88	82	76	71	70	68	65	63	60	57	54
**58%**	107	100	91	84	78	73	73	70	67	65	62	59	55
**59%**	111	103	94	87	81	75	75	72	69	67	63	60	57
**60%**	115	107	97	90	83	77	77	74	71	68	65	62	58
**61%**	119	111	101	93	86	80	79	76	74	70	67	64	60
**62%**	123	115	104	96	89	82	82	79	76	72	69	66	62
**63%**	128	119	107	99	91	84	84	81	78	75	71	67	64
**64%**	133	123	111	102	94	87	87	83	80	77	73	69	65
**65%**	137	127	115	105	97	89	89	86	82	79	75	71	67
**66%**	143	132	119	109	100	92	92	88	85	81	77	73	69
**67%**	148	136	123	112	103	95	94	91	87	83	79	75	71
**68%**	154	141	127	116	106	97	97	94	90	86	82	77	73
**69%**	159	147	131	119	109	100	100	96	92	88	84	80	75
**70%**	166	152	136	123	113	103	103	99	95	91	86	82	77
**71%**	172	157	140	127	116	106	106	102	98	93	89	84	79
**72%**	179	163	145	131	120	110	109	105	101	96	91	87	82
**73%**	186	169	150	136	124	113	113	108	103	99	94	89	84
**74%**	193	176	155	140	128	116	116	111	106	101	96	91	86
**75%**	201	183	161	145	132	120	119	114	109	104	99	94	89
**76%**	210	190	167	150	136	123	123	118	113	107	102	97	91
**77%**	218	197	172	155	140	127	127	121	116	110	105	99	94
**78%**	228	205	179	160	144	131	130	125	119	114	108	102	96
**79%**	237	213	185	165	149	135	134	128	123	117	111	105	99
**80%**	248	222	192	171	154	139	138	132	126	120	114	108	102
**81%**	259	231	199	177	159	143	143	136	130	124	117	111	105
**82%**	271	241	207	183	164	147	147	140	134	127	121	115	108
**83%**	284	251	215	190	169	152	152	145	138	131	124	118	111
**84%**	297	262	223	196	175	156	156	149	142	135	128	121	115
**85%**	312	273	232	203	181	161	161	153	146	139	132	125	118
**86%**	327	286	241	211	187	166	166	158	151	143	136	129	121
**87%**	344	299	251	219	193	172	172	163	155	147	140	132	125
**88%**	363	313	261	227	200	177	177	168	160	152	144	137	129
**89%**	382	328	273	236	207	183	183	174	165	157	148	141	133
**90%**	404	345	284	245	214	189	189	179	170	161	153	145	137
**91%**	428	363	297	254	222	195	195	185	176	167	158	150	141
**92%**	454	382	310	264	230	201	201	191	181	172	163	154	146
**93%**	483	403	325	275	238	208	208	197	187	177	168	159	151
**94%**	514	425	340	287	247	215	215	204	193	183	173	164	156
**95%**	550	450	356	299	257	222	223	211	200	189	179	170	161
**96%**	590	478	374	312	266	230	231	218	206	195	185	176	166
**97%**	635	508	393	326	277	238	239	226	214	202	191	182	172
**98%**	687	542	414	340	288	247	248	234	221	209	198	188	178
**99%**	746	579	437	356	300	256	257	242	229	216	205	194	185
**100%**	815	621	462	373	312	265	266	251	237	224	212	202	191

Supplementary Table S17. Differences between AMs' consumption levels calculated by means of novel pediatric-adjusted methodology vs conventional ATC/DDD methodology for fosfomycin (base case), %

Share / Age group	1-11 month	1 year	2 years	3 years	4 years	5 years	6 years	7 years	8 years	9 years	10 years	11 years	12 years
1%	1	1	1	1	1	0	0	0	0	0	0	0	0
2%	2	1	1	1	1	1	0	0	0	0	0	0	0
3%	2	2	2	1	1	1	0	0	0	0	0	0	0
4%	3	2	2	2	1	1	0	0	0	0	0	0	0
5%	3	3	2	2	1	1	0	0	0	0	0	0	0
6%	4	3	3	2	1	1	0	0	0	0	0	0	0
7%	5	4	3	2	2	2	1	1	1	1	1	1	1
8%	5	5	4	3	2	2	1	1	1	1	1	1	1
9%	7	5	4	3	2	2	1	1	1	1	1	1	1
10%	8	7	4	3	2	2	1	1	1	1	1	1	1
11%	8	7	5	3	2	2	1	1	1	1	1	1	1
12%	9	8	5	4	3	2	1	1	1	1	1	1	1
13%	10	8	7	4	3	2	1	1	1	1	1	1	1
14%	10	9	7	4	3	3	1	1	1	1	1	1	1
15%	11	10	7	5	3	3	1	1	1	1	1	1	1
16%	12	10	8	5	3	3	1	1	1	1	1	1	1
17%	13	11	8	5	3	3	1	1	1	1	1	1	1
18%	13	11	9	7	4	3	1	1	1	1	1	1	1
19%	14	12	9	7	4	3	1	1	1	1	1	1	1
20%	15	13	10	7	4	3	1	1	1	1	1	1	1
21%	16	13	10	8	4	3	1	1	1	1	1	1	1
22%	16	14	10	8	4	3	1	1	1	1	1	1	1
23%	17	14	11	8	5	3	1	1	1	1	1	1	1
24%	18	15	11	8	5	3	1	1	1	1	1	1	1
25%	20	16	12	9	5	3	1	1	1	1	1	1	1
26%	21	16	12	9	5	4	1	1	1	1	1	1	1
27%	22	17	13	9	5	4	1	1	1	1	1	1	1
28%	22	18	13	10	7	4	1	1	1	1	1	1	1
29%	23	18	14	10	7	4	1	1	1	1	1	1	1
30%	24	20	14	10	7	5	2	2	2	2	2	2	2
31%	25	21	15	11	7	5	2	2	2	2	2	2	2
32%	26	22	15	11	7	5	2	2	2	2	2	2	2
33%	27	22	16	11	8	5	2	2	2	2	2	2	2
34%	28	23	16	12	8	5	2	2	2	2	2	2	2
35%	29	24	17	12	8	5	2	2	2	2	2	2	2
36%	30	25	17	12	8	5	2	2	2	2	2	2	2
37%	32	25	18	13	8	5	2	2	2	2	2	2	2
38%	33	26	18	13	9	6	2	2	2	2	2	2	2
39%	34	27	20	13	9	6	2	2	2	2	2	2	2
40%	35	28	20	14	9	6	2	2	2	2	2	2	2
41%	36	29	21	14	9	6	2	2	2	2	2	2	2
42%	37	29	21	14	9	6	2	2	2	2	2	2	2
43%	38	30	22	15	10	6	2	2	2	2	2	2	2
44%	39	32	23	15	10	6	2	2	2	2	2	2	2
45%	40	33	23	16	10	6	2	2	2	2	2	2	2
46%	42	34	24	16	10	6	2	2	2	2	2	2	2
47%	43	35	24	16	10	6	2	2	2	2	2	2	2

**48%**	45	36	25	17	11	6	2	2	2	2	2	2	2
**49%**	46	36	25	17	11	7	2	2	2	2	2	2	2
**50%**	47	37	26	17	11	8	3	3	3	3	3	3	3
**51%**	49	38	27	18	11	8	3	3	3	3	3	3	3
**52%**	50	39	27	18	12	8	3	3	3	3	3	3	3
**53%**	51	40	28	20	12	8	3	3	3	3	3	3	3
**54%**	53	41	28	20	12	8	3	3	3	3	3	3	3
**55%**	54	42	29	20	12	8	3	3	3	3	3	3	3
**56%**	55	43	30	21	12	8	3	3	3	3	3	3	3
**57%**	58	45	30	21	13	8	3	3	3	3	3	3	3
**58%**	59	46	32	21	13	8	3	3	3	3	3	3	3
**59%**	61	47	33	22	13	8	3	3	3	3	3	3	3
**60%**	62	48	33	22	13	9	3	3	3	3	3	3	3
**61%**	64	49	34	23	14	9	3	3	3	3	3	3	3
**62%**	65	51	35	23	14	9	3	3	3	3	3	3	3
**63%**	67	52	35	23	14	9	3	3	3	3	3	3	3
**64%**	70	53	36	24	14	9	3	3	3	3	3	3	3
**65%**	71	54	37	24	14	9	3	3	3	3	3	3	3
**66%**	73	55	37	25	15	9	3	3	3	3	3	3	3
**67%**	75	57	38	25	15	9	3	3	3	3	3	3	3
**68%**	77	59	39	26	15	10	4	4	4	4	4	4	4
**69%**	78	60	40	26	15	10	4	4	4	4	4	4	4
**70%**	80	61	40	26	16	10	4	4	4	4	4	4	4
**71%**	83	62	41	27	16	11	4	4	4	4	4	4	4
**72%**	85	64	42	27	16	11	4	4	4	4	4	4	4
**73%**	87	65	43	28	16	11	4	4	4	4	4	4	4
**74%**	89	66	43	28	17	11	4	4	4	4	4	4	4
**75%**	91	68	45	29	17	11	4	4	4	4	4	4	4
**76%**	95	70	46	29	17	11	4	4	4	4	4	4	4
**77%**	97	72	47	30	17	11	4	4	4	4	4	4	4
**78%**	99	73	47	30	17	11	4	4	4	4	4	4	4
**79%**	101	75	48	30	18	11	4	4	4	4	4	4	4
**80%**	104	76	49	32	18	11	4	4	4	4	4	4	4
**81%**	107	78	50	32	18	11	4	4	4	4	4	4	4
**82%**	110	79	51	33	18	12	4	4	4	4	4	4	4
**83%**	112	82	52	33	20	13	5	5	5	5	5	5	5
**84%**	115	83	52	34	20	13	5	5	5	5	5	5	5
**85%**	118	85	53	34	20	13	5	5	5	5	5	5	5
**86%**	122	87	54	35	20	13	5	5	5	5	5	5	5
**87%**	124	89	55	35	21	13	5	5	5	5	5	5	5
**88%**	127	90	57	36	21	13	5	5	5	5	5	5	5
**89%**	130	92	58	36	21	13	5	5	5	5	5	5	5
**90%**	135	95	59	37	21	13	5	5	5	5	5	5	5
**91%**	138	97	60	37	22	13	5	5	5	5	5	5	5
**92%**	141	99	61	38	22	13	5	5	5	5	5	5	5
**93%**	146	101	62	38	22	14	5	5	5	5	5	5	5
**94%**	149	103	63	39	22	14	5	5	5	5	5	5	5
**95%**	153	105	64	39	23	14	5	5	5	5	5	5	5
**96%**	158	108	65	40	23	15	6	6	6	6	6	6	6
**97%**	162	110	66	40	23	15	6	6	6	6	6	6	6
**98%**	166	112	67	41	24	15	6	6	6	6	6	6	6
**99%**	171	114	68	42	24	15	6	6	6	6	6	6	6
**100%**	175	117	70	42	24	15	6	6	6	6	6	6	6

Supplementary Table S18. Differences between AMs' consumption levels calculated by means of novel pediatric-adjusted methodology vs conventional ATC/DDD methodology for antifungals (base case), %

Share / Age group	1-11 month	1 year	2 years	3 years	4 years	5 years	6 years	7 years	8 years	9 years	10 years	11 years	12 years
1%	1	1	0	0	0	0	0	0	0	0	0	0	0
2%	1	1	0	0	0	0	0	0	0	0	0	0	0
3%	2	1	1	0	0	0	0	0	0	0	0	0	0
4%	3	2	2	1	0	0	0	0	0	0	0	0	0
5%	3	2	2	1	0	0	0	0	0	0	0	0	0
6%	3	3	2	1	0	0	0	0	0	0	0	0	0
7%	4	4	2	1	0	0	0	0	0	0	0	0	0
8%	5	4	3	1	0	0	0	0	0	0	0	0	0
9%	5	4	3	2	0	0	0	0	0	0	0	0	0
10%	6	5	3	2	0	0	0	0	0	0	0	0	0
11%	7	6	4	2	1	0	0	0	0	0	0	0	0
12%	7	6	4	2	1	0	0	0	0	0	0	0	0
13%	8	6	5	2	1	0	0	0	0	0	0	0	0
14%	9	7	5	3	1	0	0	0	0	0	0	0	0
15%	9	7	5	3	1	0	0	0	0	0	0	0	0
16%	10	8	5	3	1	0	0	0	0	0	0	0	0
17%	11	9	6	3	1	0	0	0	0	0	0	0	0
18%	11	9	6	3	1	0	0	0	0	0	0	0	0
19%	12	10	7	3	1	0	0	0	0	0	0	0	0
20%	13	10	7	4	1	0	0	0	0	0	0	0	0
21%	14	11	7	4	1	0	0	0	0	0	0	0	0
22%	14	11	8	4	1	0	0	0	0	0	0	0	0
23%	15	12	8	4	1	0	0	0	0	0	0	0	0
24%	16	12	9	4	1	0	0	0	0	0	0	0	0
25%	16	13	9	5	2	1	0	0	0	0	0	0	0
26%	17	14	9	5	2	1	0	0	0	0	0	0	0
27%	18	14	10	5	2	1	0	0	0	0	0	0	0
28%	19	15	10	5	2	1	0	0	0	0	0	0	0
29%	20	15	11	5	2	1	0	0	0	0	0	0	0
30%	21	16	11	6	2	1	0	0	0	0	0	0	0
31%	21	16	12	6	2	1	0	0	0	0	0	0	0
32%	23	17	12	6	2	1	0	0	0	0	0	0	0
33%	23	18	12	6	2	1	0	0	0	0	0	0	0
34%	24	19	13	7	2	1	0	0	0	0	0	0	0
35%	25	19	13	7	2	1	0	0	0	0	0	0	0
36%	26	20	14	7	2	1	0	0	0	0	0	0	0
37%	27	21	14	7	2	1	0	0	0	0	0	0	0
38%	28	21	15	7	2	1	0	0	0	0	0	0	0
39%	29	22	15	8	2	1	0	0	0	0	0	0	0
40%	30	23	16	8	2	1	0	0	0	0	0	0	-1
41%	31	23	16	8	3	1	0	0	0	0	0	0	-1
42%	32	24	16	8	3	1	0	0	0	0	0	0	-1
43%	32	25	17	8	3	1	0	0	0	0	0	0	-1
44%	33	25	17	9	3	1	0	0	0	0	0	0	-1
45%	35	26	18	9	3	1	0	0	0	0	0	0	-1
46%	36	27	18	9	3	1	0	0	0	0	0	0	-1
47%	37	28	19	9	3	1	0	0	0	-1	-1	-1	-1

**48%**	38	28	19	9	3	1	0	0	0	-1	-1	-1	-1
**49%**	39	29	20	10	3	1	0	0	0	-1	-1	-1	-1
**50%**	40	30	20	10	3	1	0	0	0	-1	-1	-1	-1
**51%**	41	31	21	10	3	1	0	0	0	-1	-1	-1	-1
**52%**	42	32	21	11	3	1	0	0	0	-1	-1	-1	-1
**53%**	44	32	22	11	3	1	0	0	0	-1	-1	-1	-1
**54%**	45	33	22	11	3	1	0	0	0	-1	-1	-1	-1
**55%**	46	34	23	11	4	1	0	0	0	-1	-1	-1	-1
**56%**	47	35	23	11	4	1	0	0	0	-1	-1	-1	-1
**57%**	48	36	24	12	4	1	1	0	0	-1	-1	-1	-1
**58%**	50	37	25	12	4	1	1	0	0	-1	-1	-1	-1
**59%**	51	37	25	12	4	1	1	0	0	-1	-1	-1	-1
**60%**	52	38	26	12	4	1	1	0	0	-1	-1	-1	-1
**61%**	54	39	26	13	4	1	1	0	0	-1	-1	-1	-1
**62%**	55	41	27	13	4	1	1	0	0	-1	-1	-1	-1
**63%**	56	41	27	13	4	1	1	0	0	-1	-1	-1	-1
**64%**	58	42	28	13	4	1	1	0	0	-1	-1	-1	-1
**65%**	59	43	28	14	4	1	1	0	0	-1	-1	-1	-1
**66%**	61	44	29	14	4	1	1	0	0	-1	-1	-1	-1
**67%**	63	45	30	14	4	2	1	0	0	-1	-1	-1	-1
**68%**	64	46	30	14	4	2	1	0	0	-1	-1	-1	-1
**69%**	65	47	31	14	5	2	1	0	0	-1	-1	-1	-1
**70%**	67	48	31	15	5	2	1	0	0	-1	-1	-1	-1
**71%**	69	49	32	15	5	2	1	0	0	-1	-1	-1	-1
**72%**	70	50	33	15	5	2	1	0	0	-1	-1	-1	-1
**73%**	72	51	33	15	5	2	1	0	-1	-1	-1	-1	-1
**74%**	74	52	34	16	5	2	1	0	-1	-1	-1	-1	-1
**75%**	75	53	34	16	5	2	1	0	-1	-1	-1	-1	-1
**76%**	77	54	35	16	5	2	1	0	-1	-1	-1	-1	-1
**77%**	79	56	36	16	5	2	1	0	-1	-1	-1	-1	-1
**78%**	81	57	36	17	5	2	1	0	-1	-1	-1	-1	-1
**79%**	83	58	37	17	5	2	1	0	-1	-1	-1	-1	-1
**80%**	85	59	38	17	5	2	1	0	-1	-1	-1	-1	-1
**81%**	87	61	38	18	6	2	1	0	-1	-1	-1	-1	-1
**82%**	89	62	39	18	6	2	1	0	-1	-1	-1	-1	-1
**83%**	91	63	40	18	6	2	1	0	-1	-1	-1	-1	-1
**84%**	93	64	41	18	6	2	1	0	-1	-1	-1	-1	-1
**85%**	95	65	41	19	6	2	1	0	-1	-1	-1	-1	-1
**86%**	97	67	42	19	6	2	1	0	-1	-1	-1	-1	-1
**87%**	100	68	43	19	6	2	1	0	-1	-1	-1	-1	-1
**88%**	102	69	43	19	6	2	1	0	-1	-1	-1	-1	-1
**89%**	105	71	44	20	6	2	1	0	-1	-1	-1	-1	-1
**90%**	107	72	45	20	6	2	1	0	-1	-1	-1	-1	-1
**91%**	110	74	46	20	6	2	1	0	-1	-1	-1	-1	-1
**92%**	113	75	47	21	6	2	1	0	-1	-1	-1	-1	-1
**93%**	115	77	48	21	6	2	1	0	-1	-1	-1	-1	-1
**94%**	118	78	48	21	7	2	1	0	-1	-1	-1	-1	-1
**95%**	121	79	49	22	7	3	1	0	-1	-1	-1	-1	-1
**96%**	124	82	50	22	7	3	1	0	-1	-1	-1	-1	-1
**97%**	127	83	51	22	7	3	1	0	-1	-1	-1	-1	-1
**98%**	130	85	52	22	7	3	1	0	-1	-1	-1	-1	-1
**99%**	133	86	53	23	7	3	1	0	-1	-1	-1	-1	-1
**100%**	137	88	53	23	7	3	1	0	-1	-1	-1	-1	-1

Supplementary Table S19. Differences between AMs' consumption levels calculated by means of novel pediatric-adjusted methodology vs conventional ATC/DDD methodology for antivirals (base case), %

Share / Age group	1-11 month	1 year	2 years	3 years	4 years	5 years	6 years	7 years	8 years	9 years	10 years	11 years	12 years
**1%**	0	1	1	1	1	1	0	0	0	0	0	0	0
**2%**	0	1	1	1	1	1	1	1	1	1	1	1	0
**3%**	3	2	2	2	2	2	1	1	1	1	1	1	0
**4%**	3	3	3	3	3	3	2	2	2	2	1	1	0
**5%**	3	3	3	4	3	3	3	3	2	2	2	2	1
**6%**	3	4	4	4	4	4	3	3	3	3	2	2	1
**7%**	6	5	5	5	5	5	4	4	3	3	3	3	1
**8%**	6	5	6	6	5	5	4	4	3	3	3	3	1
**9%**	6	7	7	7	6	6	5	5	4	4	3	3	1
**10%**	10	8	8	7	7	7	5	5	5	4	4	4	1
**11%**	10	8	9	8	8	8	6	6	5	5	4	4	1
**12%**	10	9	9	9	8	8	7	6	5	5	5	4	1
**13%**	13	10	10	10	9	9	7	7	6	6	5	5	2
**14%**	13	11	11	11	10	10	8	7	7	6	6	5	2
**15%**	13	11	12	12	11	11	8	8	7	7	6	6	2
**16%**	16	12	13	13	12	11	9	8	7	7	7	6	2
**17%**	16	13	14	13	12	12	10	9	8	8	7	7	2
**18%**	19	14	15	15	13	13	10	10	8	8	7	7	2
**19%**	19	15	16	15	14	14	11	10	9	9	8	8	3
**20%**	19	16	17	16	15	15	12	11	9	9	8	8	3
**21%**	23	17	18	17	16	16	12	12	10	10	9	8	3
**22%**	23	18	18	18	17	17	13	12	11	10	9	9	3
**23%**	26	19	20	19	18	17	13	12	11	11	10	9	3
**24%**	26	20	21	20	19	18	14	13	11	11	10	10	3
**25%**	29	21	22	21	20	19	15	14	12	12	10	10	3
**26%**	29	22	23	23	20	20	16	14	13	12	11	11	3
**27%**	29	23	24	24	22	21	16	15	13	13	12	11	4
**28%**	32	24	25	24	22	22	17	16	14	14	12	12	4
**29%**	32	25	26	26	23	23	18	16	14	14	12	12	4
**30%**	35	26	28	27	25	24	19	17	15	15	13	13	4
**31%**	35	28	29	28	26	25	20	18	16	15	14	13	4
**32%**	39	29	30	29	27	26	20	18	16	16	14	13	4
**33%**	39	30	31	30	28	27	21	19	17	16	14	14	4
**34%**	42	31	33	32	29	28	22	20	18	17	15	14	5
**35%**	45	32	34	33	30	29	23	21	18	18	16	15	5
**36%**	45	34	35	34	31	31	23	21	19	18	16	16	5
**37%**	48	35	36	35	32	32	24	22	19	19	16	16	5
**38%**	48	36	38	37	33	33	25	23	20	19	17	16	5
**39%**	52	37	39	38	35	34	26	24	21	20	18	17	5
**40%**	55	39	41	39	36	35	27	24	21	21	18	17	5
**41%**	55	40	42	41	37	37	28	25	22	21	19	18	6
**42%**	58	41	43	42	38	38	28	26	23	22	19	19	6
**43%**	61	43	45	44	40	39	29	27	24	23	20	19	6
**44%**	61	44	47	45	41	40	30	27	24	23	21	20	6
**45%**	65	46	48	47	43	42	31	28	25	24	21	20	6
**46%**	68	47	50	48	44	43	32	29	25	25	22	21	6
**47%**	71	49	52	50	45	45	33	30	26	26	22	21	7

**48%**	74	51	53	51	47	46	34	31	27	26	23	22	7
**49%**	74	52	55	53	48	47	35	32	28	27	24	23	7
**50%**	77	54	57	55	50	49	36	33	29	28	24	23	7
**51%**	81	56	59	57	51	50	37	33	29	28	25	24	7
**52%**	84	58	60	58	53	52	38	34	30	29	26	25	8
**53%**	87	60	62	60	55	54	39	35	31	30	26	25	8
**54%**	90	61	64	62	56	55	40	36	32	31	27	26	8
**55%**	94	63	66	64	58	57	41	37	32	31	27	26	8
**56%**	97	65	68	66	59	58	42	38	33	32	28	27	8
**57%**	100	67	71	68	61	60	43	39	34	33	29	28	8
**58%**	106	69	73	70	63	62	45	40	35	34	29	28	8
**59%**	110	71	75	72	65	64	46	41	36	35	30	29	9
**60%**	113	74	77	74	67	66	47	42	36	35	31	29	9
**61%**	116	76	80	76	69	67	48	43	37	36	31	30	9
**62%**	123	78	82	78	71	70	49	44	38	37	32	31	9
**63%**	126	80	84	81	73	71	50	45	39	38	33	32	9
**64%**	129	82	87	83	75	73	51	46	40	39	34	32	9
**65%**	135	85	90	86	77	76	53	47	41	40	35	33	10
**66%**	139	87	92	88	80	78	54	48	42	41	35	34	10
**67%**	145	91	95	91	82	80	56	49	43	41	36	34	10
**68%**	152	93	98	93	84	83	57	50	44	42	37	35	10
**69%**	158	96	101	96	86	85	58	51	45	43	37	36	10
**70%**	165	99	104	99	89	87	60	53	46	44	38	37	11
**71%**	171	102	107	102	92	90	61	54	47	45	39	37	11
**72%**	177	105	110	105	94	92	62	55	48	46	40	38	11
**73%**	184	108	114	108	97	95	64	56	49	47	41	39	11
**74%**	190	112	117	111	100	97	66	57	50	48	41	40	11
**75%**	197	115	121	114	103	100	67	59	51	49	42	41	11
**76%**	206	118	124	118	106	103	69	60	52	50	43	41	12
**77%**	216	122	128	121	109	106	70	61	53	51	44	42	12
**78%**	226	126	132	125	112	109	72	63	54	52	45	43	12
**79%**	235	130	136	128	115	113	74	64	55	53	46	44	12
**80%**	245	134	141	132	119	116	76	65	57	54	47	45	12
**81%**	255	138	145	136	122	119	77	67	58	56	48	46	13
**82%**	268	143	150	141	126	123	79	68	59	57	49	46	13
**83%**	281	147	155	145	130	126	81	70	60	58	50	47	13
**84%**	294	153	160	149	134	130	83	71	61	59	51	48	13
**85%**	306	158	165	154	138	134	85	73	63	60	52	49	13
**86%**	323	163	170	159	142	138	87	74	64	61	53	50	14
**87%**	339	169	176	164	146	142	89	76	66	63	54	51	14
**88%**	358	175	182	169	151	147	91	77	67	64	55	52	14
**89%**	377	181	188	174	156	151	93	79	68	65	56	53	14
**90%**	400	187	195	180	161	156	95	81	70	67	57	54	14
**91%**	423	194	202	186	166	161	98	82	71	68	58	55	14
**92%**	448	202	209	192	171	166	100	84	72	69	59	56	15
**93%**	477	209	216	198	177	172	102	86	74	70	60	57	15
**94%**	510	218	224	205	183	177	105	87	75	72	61	58	15
**95%**	542	226	233	212	189	183	107	89	77	73	62	59	15
**96%**	584	236	241	219	195	189	110	91	78	75	63	60	16
**97%**	626	247	251	227	202	196	112	93	80	76	65	62	16
**98%**	677	257	261	235	209	203	115	95	82	78	66	63	16
**99%**	735	269	272	244	217	210	118	97	83	79	67	64	16
**100%**	803	282	283	253	225	218	121	99	85	81	68	65	17

Supplementary Table S20. Doses and dosing approaches from the national summaries of product characteristics [3] used to calculate cDDDs of AMs for children aged 1 month to 12 years

INN	Adm.R*	ATC code	Daily dose (range)			
			based on weight, g/kg	based on age, g	based on BSA, g/m^2	other
Azithromycin	O	J01FA10	0.01 (0.01-0.02)			
Azithromycin	P	J01FA10	NA	NA	NA	NA
Aztreonam	P	J01DF01	0.12 (0.09-0.12)			
Amikacin	P	J01GB06	0.02 (0.015-0.02)			
Amoxicillin	O	J01CA04	0.06 (0.03-0.06)			
Amoxicillin and beta-lactamase inhibitor	O	J01CR02	0.06 (0.03-0.06)			
Amoxicillin and beta-lactamase inhibitor	P	J01CR02	0.1 (0.075-0.1)			
Ampicillin	P	J01CA01	0.2 (0.1-0.2)			
Ampicillin and beta-lactamase inhibitor	P	J01CR01	0.2 (0.1-0.2)			
Amphotericin B	P	J02AA01	0.0015 (0.0003-0.0015)			
Anidulafungin	P	J02AX06	0.0015 (ND)			
Acyclovir	O	J05AB01	0.08 (ND)			
Acyclovir	P	J05AB01	0.06 (ND)			
Benzathine Benzylpenicillin	P	J01CE08	<30 kg - ½ adult DDD; ≥30 kg - adult DDD			
Benzylpenicillin	P	J01CE01	<25 kg – ½ adult DDD; ≥25 kg - adult DDD			
Bicillin-5	P	J01CE30		≤7 years – 1.1 g/day; ≥8 years – 2.2 g/day		
Valaciclivir	O	J05AB11	NA	NA	NA	NA
Valganciclovir	O	J05AB14	NA	NA	NA	NA
Vancomycin	O	A07AA09	0.04 (ND)			
Vancomycin	P	J01XA01	0.04 (ND)			
Voriconazole	O	J02AC03	0.016 (ND)			
Voriconazole	P	J02AC03	0.018 (ND)			
Ganciclovir	O	J05AB06	NA	NA	NA	NA
Ganciclovir	P	J05AB06	NA	NA	NA	NA
Gentamicin	P	J01GB03	0.005 (0.003-0.005)			
Griseofulvin	O	D01BA01	0.01 (ND)			
Dalbavantsin	P	J01XA04	NA	NA	NA	NA
Daptomycin	P	J01XX09		1-2 year - 0.01 g/day; 2-6 years - 0.009 g/day; 7-11 years - 0.007 g/day; 12 years - 0.005 g/day		
Josamycin	O	J01FA07	0.05 (0.04-0.05)			
Doxycycline	O	J01AA02	0.004 (0.002-0.004)			
Doxycycline	P	J01AA02	0.004 (0.002-0.004)			
Doripenem	P	J01DH04	NA	NA	NA	NA
Zanamivir	O	J05AH01				0.02 g/day
Isavuconazole	O	J02AC05	NA	NA	NA	NA

Isavuconazole	P	J02AC05	NA	NA	NA	NA
Imipenem and cilastatin	P	J01DH51	0.06 (ND)			
Itraconazole	O	J02AC02	0.005 (ND)			
Kanamycin	P	J01GB04	0.015 (ND)			
Caspofungin	P	J02AX04			0.07 (0.05-0.07)	
Ketoconazole	O	J02AB02	<30 kg - 0.1 g/day; ≥30 kg - 0.2 g/day			
Clarithromycin	O	J01FA09	0.015 (ND)			
Clarithromycin	P	J01FA09	NA	NA	NA	NA
Clindamycin	O	J01FF01				0.6-1.8 g/day
Clindamycin	P	J01FF01	0.04 (0.02-0.04)			
Co-Trimoxazole	O	J01EE01	0.036 (ND)			
Co-Trimoxazole	P	J01EE01	0.036 (ND)			
Levofloxacin	O	J01MA12	NA	NA	NA	NA
Levofloxacin	P	J01MA12	NA	NA	NA	NA
Linezolid	O	J01XX08	0.03 (ND)			
Linezolid	P	J01XX08	0.03 (ND)			
Lincomycin	O	J01FF02	0.06 (0.03-0.06)			
Lincomycin	P	J01FF02	0.02 (0.01-0.02)			
Lomefloxacin	O	J01MA07	NA	NA	NA	NA
Meropenem	P	J01DH02	0.12 (0.03-0.12)			
Metronidazole	O	P01AB01				0.5 g/day
Metronidazole	P	J01XD01	0.0225 (ND)			
Midecamycin	O	J01FA03	0.05 (0.02-0.05)			
Micafungin	P	J02AX05	0.004 (0.002-0.004)			
minocycline	O	J01AA08	0.004 (0.002-0.004)			
Moxifloxacin	O	J01MA14	NA	NA	NA	NA
Moxifloxacin	P	J01MA14	NA	NA	NA	NA
Natamycin	O	A07AA03				0.2 g/day
Netilmicin	P	J01GB07	0.0075 (0.006-0.0075)			
Nimorazole	O	P01AB06	0.02 (0.015-0.02)			
Nystatin	O	A07AA02				1 g/day
Nitroxoline	O	J01XX07		3-4 years - 0.2 g/day; ≥5 years - 0.2-0.4 g/day		
Nitrofurantoin	O	J01XE01	0.003 (ND)			
Nifuratel	O	G01AX05	NA	NA	NA	NA
Nifuroxazide	O	A07AX03		<3 years - 0.3 g/day; ≥3 years - 0.6 g/day		
Norfloxacin	O	J01MA06	NA	NA	NA	NA
Oxacillin	P	J01CF04		<2 years - 1 g/day; ≥2 years - 2 g/day		
Ornidazole	O	P01AB03	0.04 (0.025-0.04)			
Ornidazole	P	J01XD03	0.03 (0.02-0.03)			
Oseltamivir	O	J05AH02	<15 kg - 0.06 g/day; 15-23 kg - 0.09 g/day; >23-40 kg - 0.12 g/day			
Ofloxacin	O	J01MA01	NA	NA	NA	NA

Ofloxacin	P	J01MA01	NA	NA	NA	NA
Pefloxacin	O	J01MA03	NA	NA	NA	NA
Pefloxacin	P	J01MA03	NA	NA	NA	NA
Pipemidic acid	O	J01MB04	NA	NA	NA	NA
Piperacillin and beta-lactamase inhibitor	P	J01CR05	0.3 (ND)			
Posaconazole	O	J02AC04	NA	NA	NA	NA
Polymyxin B	P	J01XB02	0.0025 (0.0015-0.0025)			
Rimantadine	O	J05AC02		<3 years - 0.04 g/day; 3-7 years - 0.06 g/day; 7-10 years - 0.1 g/day; 10-12 years - 0.15 g/day		
Rifaximin	O	A07AA11				0.6-0.8 g/day
Roxithromycin	O	J01FA06	0.008 (ND)			
Secnidazole	O	P01AB07	0.03 (ND)			
Sparfloxacin	O	J01MA09	NA	NA	NA	NA
Spectinomycin	P	J01XX04	0.04 (ND)			
Spiramycin	O	J01FA02	0.09 (0.045-0.09); max 2.8 g/day			
Streptomycin	P	J01GA01	0.02 (0.015-0.02)			
Sultamicillin	O	J01CR04	0.05 (0.025-0.05); max 0.75 g/day			
Sulfadimethoxine	O	J01ED01	0.0125 (0.0125-0.025)			
Sulfadimidine	O	J01EB03	0.15 (0.05-0.15)			
Tedizolid	O	J01XX11	NA	NA	NA	NA
Tedizolid	P	J01XX11	NA	NA	NA	NA
Teicoplanin	P	J01XA02	0.01 (0.006-0.01)			
Terbinafine	O	D01BA02				0.125 g/day
Tetracycline	O	J01AA07	0.1 (0.05-0.1)			
Thiamphenicol	P	J01BA02		<3 years - 0.25 g/day; 3-7 years - 0.5 g/day; 7-12 years - 0.75 g/day		
Tigecycline	P	J01AA12	0.0024 (ND)			
Tinidazole	O	P01AB02	0.075 (0.05-0.075)			
Umifenovir	O	J05AX13		2-6 years - 0.2 g/day; 6-11 years - 0.4 g/day; 12 years - 0.8 g/day		
Favipiravir	O	J05AX27	NA	NA	NA	NA
Famciclovir	O	J05AB09	NA	NA	NA	NA
Fluconazole	O	J02AC01	0.012 (0.003-0.012)			
Fluconazole	P	J02AC01	0.012 (0.003-0.012)			
Fosfomycin	O	J01XX01				2 g/day
Fosfomycin	P	J01XX01	0.4 (0.2-0.4)			

Fusidic acid	O	J01XC01	3-4 years – 40-60 mg/kg; >4 years – 20-40 mg/kg			
Furazidin	O	J01XE03	NA	NA	NA	NA
Furazolidone	O	G01AX06	0.01 (0.006-0.01)			
Chloramphenicol	O	J01BA01	0.05 (0.0375-0.05)			
Cefazolin	P	J01DB04	0.1 (0.025-0.1)			
Cefalexin	O	J01DB01	0.1 (0.025-0.1)			
Cefamandol	P	J01DC03	0.15 (0.05-0.15)			
Cefditoren	O	J01DD16				0.4-0.8 g/day
cefepime	P	J01DE01	0.15 (ND)			
Cefixime	O	J01DD08	0.008 (ND)			
Cefoxitin	P	J01DC01	0.16 (0.09-0.16)			
Cefoperazone	P	J01DD12	0.2 (0.05-0.2)			
Cefoperazone and beta-lactamase inhibitor	P	J01DD62	0.08 (0.02-0.08)			
Cefotaxime	P	J01DD01	0.1 (0.05-0.1)			
Cefpir	P	J01DE02				2-4 g/day
Ceftazidime	P	J01DD02	0.15 (0.1-0.15)			
Ceftazidime and beta-lactamase inhibitor	P	J01DD52	NA	NA	NA	NA
Ceftaroline	P	J01DI02	<1 year - 0.024 mg/kg; ≥2 years - 0.036 mg/kg; max - 1.2 g/day			
Ceftolosan and beta-lactamase inhibitor	P	J01DI54	NA	NA	NA	NA
Ceftriaxone	P	J01DD04	0.1 (0.02-0.1)			
Cefuroxime	P	J01DC02	0.1 (0.03-0.1)			
Cefuroxime	O	J01DC03	0.015 (0.01-0.015)			
Ciprofloxacin	O	J01MA02	0.04 (ND)			
Ciprofloxacin	P	J01MA02	0.03 (ND)			
Erythromycin	O	J01FA01	NA	NA	NA	NA
Erythromycin	P	J01FA01	0.05 (0.03-0.05)			
Ertapenem	P	J01DH03	0.03 (ND)			

NA - not applicablem, ND – no data

Reference:

1. WHO Collaborating Centre for Drug Statistics Methodology. *Guidelines for ATC classification and DDD assignment 2023*. Oslo, Norway, 2022.
2. World Health Organisation. Child growth standards. Available online: https://www.who.int/tools/child-growth-standards (accessed on 01.06.2023)
3. Russian State Register of Medicines. Available online: https://grls.rosminzdrav.ru/material/national/medicine-registry (accessed on 01.06.2023)
